# Combination of acamprosate and baclofen (PXT864) as a potential new therapy for amyotrophic lateral sclerosis

**DOI:** 10.1002/jnr.24714

**Published:** 2020-08-19

**Authors:** Lydie Boussicault, Julien Laffaire, Peter Schmitt, Philippe Rinaudo, Noëlle Callizot, Serguei Nabirotchkin, Rodolphe Hajj, Daniel Cohen

**Affiliations:** ^1^ PHARNEXT Paris 75009 France; ^2^ NEUROSYS SAS Gardanne France

**Keywords:** amyotrophic lateral sclerosis, neuroprotection, RRID:AB_143157, RRID:AB_2271509, RRID:AB_477193, RRID:AB_477257, RRID:AB_477272, RRID:AB_2633275, SOD1, TDP‐43, therapy

## Abstract

There is currently no therapy impacting the course of amyotrophic lateral sclerosis (ALS). The only approved treatments are riluzole and edaravone, but their efficacy is modest and short‐lasting, highlighting the need for innovative therapies. We previously demonstrated the ability of PXT864, a combination of low doses of acamprosate and baclofen, to synergistically restore cellular and behavioral activity in Alzheimer's and Parkinson's disease models. The overlapping genetic, molecular, and cellular characteristics of these neurodegenerative diseases supported investigating the effectiveness of PXT864 in ALS. As neuromuscular junction (NMJ) alterations is a key feature of ALS, the effects of PXT864 in primary neuron‐muscle cocultures injured by glutamate were studied. PXT864 significantly and synergistically preserved NMJ and motoneuron integrity following glutamate excitotoxicity. PXT864 added to riluzole significantly improved such protection. PXT864 activity was then assessed in primary cultures of motoneurons derived from SOD1^G93A^ rat embryos. These motoneurons presented severe maturation defects that were significantly improved by PXT864. In this model, glutamate application induced an accumulation of TDP‐43 protein in the cytoplasm, a hallmark that was completely prevented by PXT864. The anti‐TDP‐43 aggregation effect was also confirmed in a cell line expressing TDP‐43 fused to GFP. These results demonstrate the value of PXT864 as a promising therapeutic strategy for the treatment of ALS.

## Graphical abstract

The activity of PXT864, a combination of acamprosate and baclofen, was assessed in cellular models relevant for ALS disease. PXT864 protected neuro muscular junctions in co‐cultures of primary rat neurons and human muscle cells, as well as motoneurons derived from SOD1^G93A^ rodents against glutamate toxicity. When combined with riluzole, PXT864 exerted larger protective effects, highlighting the potential use of PXT864 with the standard of care. Importantly, PXT864 reduced TDP‐43 toxic accumulation in cells after insulting stimuli such as glutamate or oxidative stress inducers. An improved maturity of cultured primary SOD1^G93A^ motoneurons was also found after PXT864 treatment.

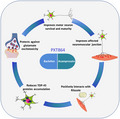

**Significance**

We describe a positive activity of a novel combination of two drugs—acamprosate and baclofen, namely PXT864—on a set of endpoints in relevant models of amyotrophic lateral sclerosis (ALS). We show that PXT864 protects neuromuscular junctions and motoneurons *in vitro*. PXT864 prevents the accumulation of toxic proteins such as TDP‐43, a major hallmark of the disease. When PXT864 is added to riluzole treatment, protection against glutamate‐induced damages is even improved. We provided the rationale for further testing of PXT864 in animal models of ALS or direct translation in patients, and a proof of principle for the value of repurposing approved and clinically safe drugs, using reproducible preclinical systems of ALS.

## 1 INTRODUCTION

Amyotrophic lateral sclerosis (ALS) is a debilitating and fatal disease characterized by the progressive degeneration of upper and lower motoneurons leading to muscle weakness and paralysis. One hallmark of the disease in human patients is cytoplasmic accumulation and aggregation of the RNA‐binding protein TDP‐43 in the brain and spinal cord (Neumann et al., 2006), indicating an important role of RNA metabolism and transport dysregulation. Several genetic factors have been identified that drive or modify motoneuron degeneration in ALS (Cirulli et al., 2015; Taylor, Brown, & Cleveland, 2016). The first mutation described for causing familial ALS affects the ubiquitously expressed cytoplasmic enzyme Cu‐Zn superoxide dismutase (SOD1), which is involved in reactive oxygen species detoxification (Rosen et al., 1993). Overexpression of mutated SOD1 (SOD1^G93A^) in transgenic mice induced motoneuron degeneration (Gurney et al., 1994). This model has been extensively studied and has revealed pathological mechanisms involved in ALS. Among them, dysfunction and degeneration of neuromuscular junctions (NMJs) was shown to precede motoneuron death in SOD1^G93A^ transgenic mice and ALS patients (Dadon‐Nachum, Melamed, & Offen, 2011; Rocha, Pousinha, & Correia, Sebastião, & Ribeiro, 2013), highlighting the crucial role of NMJ alterations in the disease.

Despite identification of such pathological mechanisms, there are still no neuroprotective therapies that significantly impact the course of the disease, and patients die on average within 3–5 years of disease onset. The only approved treatments for ALS are riluzole and edaravone, but their efficacy is modest and short‐lasting, highlighting the urgent need for innovative therapies (Fang et al., 2018; Hogg, Halang, Woods, Coughlan, & Prehn, 2017; Lacomblez, Bensimon, Leigh, Guillet, & Meininger, 1996).

Given the multifactorial and complex molecular nature of ALS, poly‐therapeutic interventions could be considered as the most promising approaches (Attarian et al., 2014; Chumakov et al., 2014). We previously demonstrated the ability of a combination therapy (PXT864), consisting of low doses of acamprosate (ACP) and baclofen (BCL), to synergistically restore cellular and behavioral endpoints affected in Alzheimer's (AD) and Parkinson's disease (PD) models (Chumakov et al., 2015; Hajj et al., 2015). AD, PD, and ALS share common cellular and molecular features. Among them, glutamate‐induced excitotoxicity is considered a major pathophysiological factor contributing to neuronal death. Altered excitatory neurotransmission has been reported in all forms of ALS, resulting in aberrant spontaneous discharge of lower motoneurons onto the muscle fibers they innervate (King, Woodhouse, Kirkcaldie, & Vickers, 2016). Other common aspects point to the excitatory and inhibitory balance that seems to be dysregulated in these diseases (Cheah, Vucic, Krishnan, & Kiernan, 2010; Do‐Ha, Buskila, & Ooi, 2018; Giovannetti & Fuhrmann, 2019; King et al., 2016; Ramírez‐Jarquín & Tapia, 2018; Sanjari Moghaddam, Zare‐Shahabadi, Rahmani, & Rezaei, 2017). We therefore hypothesized that regulation of glutamatergic and GABAergic imbalance may be beneficial in ALS. In light of this hypothesis and previous successful interventions in AD and PD with PXT864 (Chumakov et al., 2015; Hajj et al., 2015), we investigated the effectiveness of this poly‐therapeutic intervention for ALS. To this end, we assessed the activity of the combination (PXT864) of ACP and BCL in *in vitro* models relevant to ALS pathology. We took advantage of an *in vitro* NMJ model to investigate neuroprotective properties of PXT864 against glutamate excitotoxicity. Furthermore, primary motoneuron cultures derived from SOD1^G93A^ rats were used to assess PXT864 action on neuronal protection and maturation in the presence and absence of glutamate insults. The activity of PXT864 on TDP‐43 accumulation and aggregation was also studied in SOD1^G93A^ rat motoneurons and U2OS human cells line overexpressing TDP‐43 respectively. The nonantagonistic activity between PXT864 and riluzole was also assessed *in vitro* in anticipation for future clinical trials where PXT864 could be added to the standard of care.

## 2 MATERIALS AND METHODS

### 2.1 Ethics

Animal care was conducted in accordance with Directive 2010/63/UE of the European Community Council. All experiments and protocols were authorized and approved by the Ministère de l'Enseignement supérieur, de la Recherche et de l'Innovation (MESRI), as well as by local animal welfare committees Neuronexperts and Neuro‐Sys.

### 2.2 Animals

For *in vitro* experiments, pregnant female Wistar (pregnancy time = E13 for nerve/muscle cells coculture and E15 for cortical neurons primary culture) and SOD1^G93A^ rats (pregnancy time = E14) were purchased from Janvier Labs (France) and Taconic bioscience (USA), respectively. Animals were euthanized using a deep anesthesia with CO_2_ chamber and a cervical dislocation. To determine SOD1^G93A^ genotype of embryos, DNA was extracted using the SYBR Green Extract‐N‐Amp tissue PCR kit (Sigma‐Aldrich) and a qPCR was performed using the SYBR Green Master Mix kit (Sigma‐Aldrich) and human SOD1‐specific primers SOD‐i3f (GTGGCATCAGCCCTAATCCA) and SOD‐E4r (CACCAGTGTGCGGCCAATGA).

### 2.3 Drugs

(RS)‐baclofen (BCL, cat#B5399), acamprosate calcium (ACP, cat#A6981), riluzole (cat#R116), and glutamate (cat#G1501) were provided by Sigma‐Aldrich. Drugs were solubilized in 0.1% dimethyl sulfoxide (DMSO, Pan Biotech).

### 2.4 Cell cultures and treatment

Experiments were performed at Neuronexpert (France) and Neuro‐Sys (France) (Details on data origins are available in Table 1).

**TABLE 1:** Origins of data

Figures	Model	Origin of the data
**1**	Nerve/muscle coculture	Neuronexpert (France) and Neuro‐Sys (France)
**2**	Nerve/muscle coculture	Neuronexpert (France) and Neuro‐Sys (France)
**3**	Primary motoneuron culture (SOD1^G93A^ rat)	Neuro‐Sys (France)
**4**	Primary motoneuron culture (SOD1^G93A^ rat)	Neuro‐Sys (France)
**5**	U2OS expressing turbo‐GFP‐TDP‐43 cell line	Innoprot (Spain)
**6**	Primary cortical neurons	Neuronexpert (France)

#### 2.4.1 Primary neuron‐muscle cocultures

Primary neuron‐muscle cocultures were performed as previously described (Combes, Poindron, & Callizot, 2015). The human muscle cell line was purchased from Promocell (cat#C‐12530) and was plated (21 000 cells per well) in gelatin‐coated 1% (Sigma‐Aldrich, Cat#G9382) in water on 48‐well plate and grown in a proliferation medium consisting of mix of 62% of MEM medium (Minimum Essential Media, Pan Biotech, Cat#P04‐08500) and 25% of M199 medium (Pan Biotech, Cat#P04‐07500) supplemented with glutamine 2 mM (Pan Biotech, Cat#P04‐80100), human insulin 10 µg/ml (Pan Biotech, Cat#P07‐04100), human recombinant epidermal growth factor 10 ng/ml (EGF, Gibco, Cat#PHG0311), human recombinant Fibroblast growth factor basic 2 ng/ml (bFGF, Pan Biotech, Cat#CB‐1102024), fetal calf serum 10% (FCS, Gibco, Cat#10270106), and 2% of penicillin 10,000 U/ml and streptomycin 10 g/ml (PS, Pan Biotech, Cat#P06‐07100). The medium was changed every 2 days. Five days after the start of culture, immediately after satellite cell fusion, whole transverse slices of 13‐day‐old rat Wistar embryos' spinal cords with four dorsal root ganglia (DRG) attached were placed on the muscle monolayer (one explant per well in the central area). DRG were necessary to achieve a good ratio of innervation. Innervated cultures were maintained in a mixed (67% MEM/25% medium 199) medium composed of MEM and medium 199, supplemented with 5% FCS, insulin 5 µg/ml, 1% glutamine 2 mM, and 2% PS. After 24 hr of coculture, neurites were observed growing out of the spinal cord explants. These neurites made contacts with myotubes and induced the first contractions after ~8 days.

**FIGURE 1:**
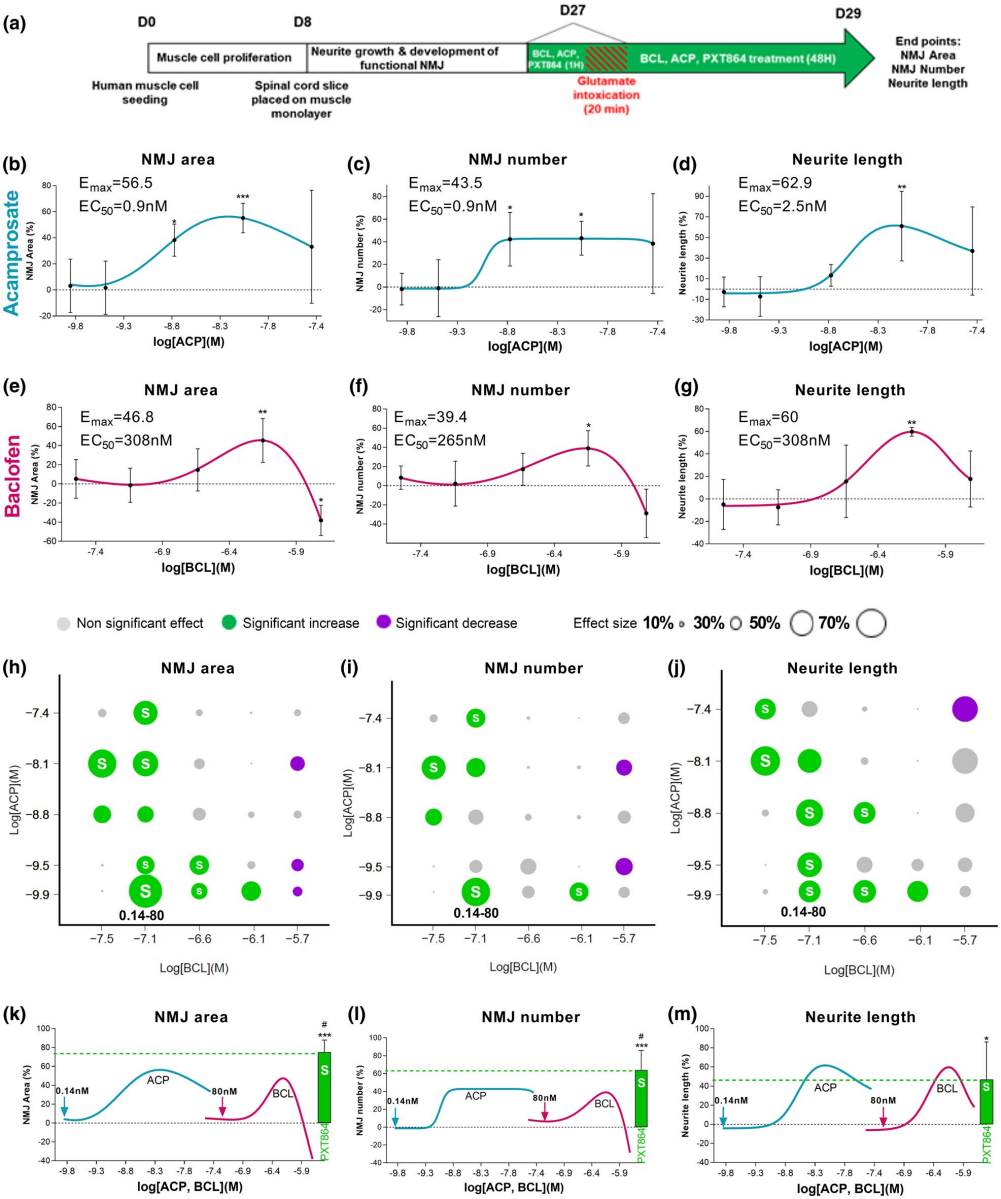
Combinations of acamprosate and baclofen act synergistically to protect neuron‐muscle cocultures against glutamate toxicity. (a) Schematic of neuron‐muscle coculture treatments. WT Wistar E13 embryos and human muscle cell line were used to generate neuron‐muscle coculture. ACP and BCL showed dose‐dependent protective properties against glutamate toxicity as measured by total NMJ area (b and e), total NMJ number (c and f), and neurite length (d and g). PXT864 combinations were tested for protective activity against glutamate, as measured by NMJ area (h), NMJ number (i), and neurite length (j). Significant increases in protection are represented by green circles, nonsignificant changes by grey circles, and significant decreases by purple circles. Circle diameter represents the effect size of the combination, and synergistic combinations are shown with a white S. Association of subactive doses of ACP (0.14 nM) and BCL (80 nM) had a synergistic activity significantly higher than maximal effects (E_max_) of each drug alone on NMJ area and number (k, l), but equal for neurite length (m). Green arrows represent drug doses (ACP 0.14 nM and BCL 80 nM) combined in PXT864 (green bar). In control condition (without any treatment), value of 100 is equivalent to 2,092 µm^2^ ± 89 for NMJ area or to 39 ± 1.59 NMJ number (per field). Data are presented as mean ± *SD*. *p < 0.05; **p < 0.01; and ***p < 0.001; treatment versus glutamate condition assessed by ANOVA with Dunnett's test (b: *F*(5,29) = 6.725, *p* = 0.0003; c: *F*(5,29) = 4.50, *p* = 0.0037; d: *F*(5,26) = 5.332, *p* = 0.0017; e: *F*(5,28) = 10.19, *p* < 0.0001; f: *F*(5,28) = 5.297, *p* = 0.0015; g: *F*(5,24) = 5.586, *p* = 0.0015). ^#^ *p* < 0.05; PXT864 versus ACP and BCL E_max_ assessed by *t*‐test. Neuron‐muscle coculture data were derived from five independent cultures with six replicates per experiment [Color figure can be viewed at wileyonlinelibrary.com]

**FIGURE 2:**
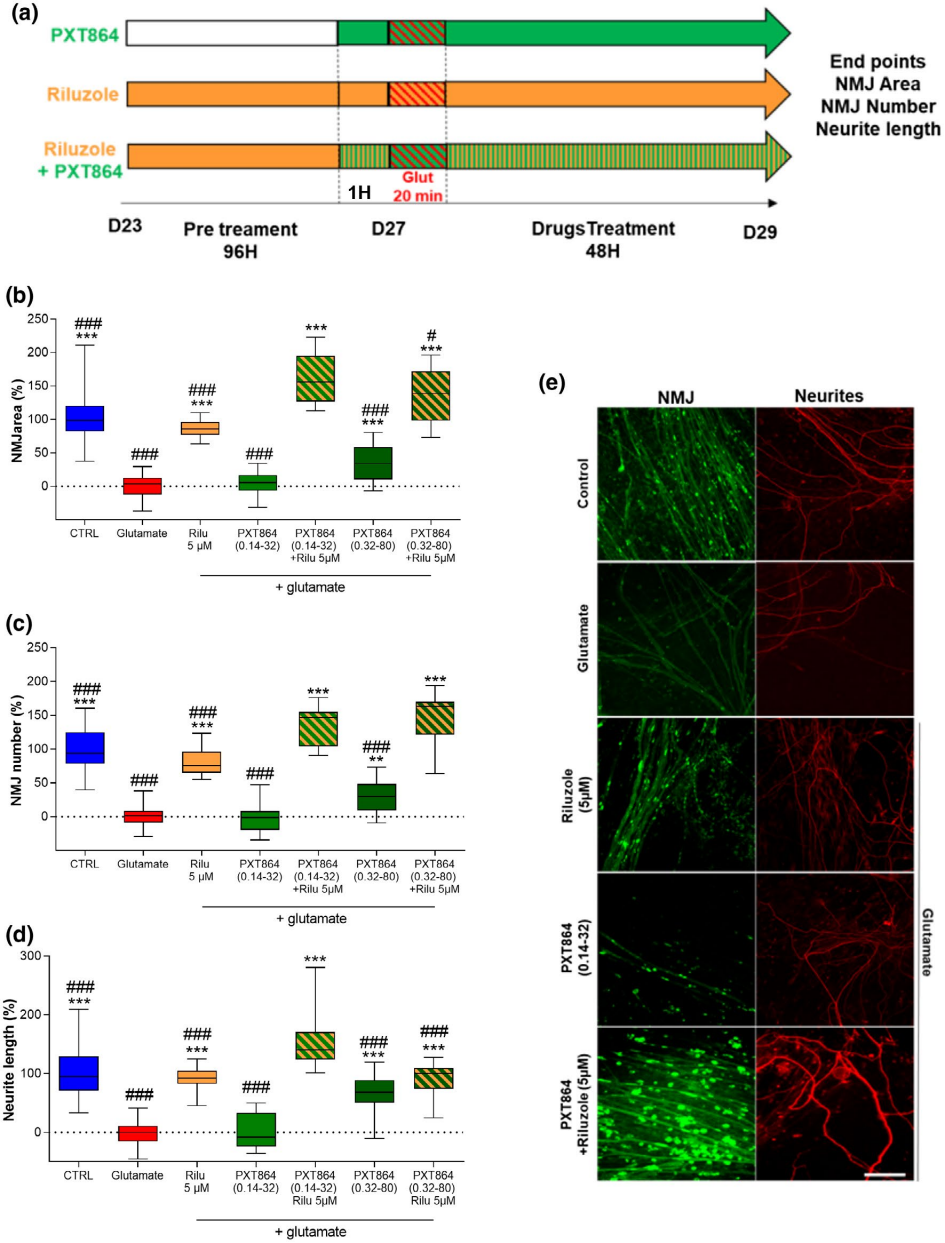
PXT864 substantially improves the protection of neuron‐muscle cocultures against glutamate toxicity under continuous riluzole treatment. (a) Schematic of neuron muscle coculture treatment with riluzole. WT Wistar E13 embryos and human muscle cell line were used to generate neuron‐muscle coculture. Protective effects of PXT864 and riluzole, alone or in combination, against glutamate toxicity were assessed by NMJ area (b), number (c), and neurite length (d, e). PXT864 (ACP 0.14 nM, BCL 32 nM) did not show significant activity when applied alone while combination with riluzole increased its protective properties on NMJ area (b), number (c), and neurite length (d). Same effect was observed for the active PXT864 (ACP 0.32 nM, BCL 80 nM). In control condition (without any treatment), value of 100 is equivalent to 1,175µm^2^ ± 38 for NMJ area or to 15 ± 0.82 NMJ number (per field). Data are presented as min to max box plots. *p < 0.05; **p < 0.01; and ***p < 0.001; treatment versus glutamate condition, and ^##^ *p* < 0.01; ^###^ *p* < 0.001; treatment versus PXT864 (0.14–32) and riluzole, assessed by ANOVA with Dunnett's test (b: *F*(6, 138) = 100.4, *p* < 0.0001; c: *F*(6, 138) = 115.7, *p* < 0.0001 and d: *F*(6, 136) = 56.82, *p* < 0.0001). Scale bar = 200 µm (e). Neuron‐muscle coculture data were derived from five independent experiments with six replicates per experiment [Color figure can be viewed at wileyonlinelibrary.com]

On day 27 after the beginning of the culture, contracting cocultures were preincubated for 1 hr with either ACP (0.14 nM to 40 nM), BCL (32 nM to 2000 nM), or PXT864 (25 combinations) before glutamate intoxication (60 μM for 20 min), and for a further 48 hr (Figure 1a). To check the contractile functionality of the NMJs and to avoid any confusion with spontaneous contractions, the cocultures were treated with acetyl choline (100 nM). Only cocultures that immediately after application, showed contractions were retained for the study and distributed into the treatment groups. The effects of riluzole on the same endpoints below were investigated. On day 23, cultures were preincubated for 96 hr with or without riluzole (5 µM) followed by incubation with PXT864 (0.14 nM ACP and 32 nM BCL), riluzole, or both 1 hr prior to glutamate intoxication and for a further 48 hr (Figure 2a).

#### 2.4.2 Primary motoneuron cultures

SOD1^G93A^ rat spinal cords were cultured as previously described (Martinou, Martinou, & Kato, 1992; Wang et al., 2013) with modification. Briefly, embryos (E14) were collected and immediately placed in ice‐cold L15 Leibovitz medium (Dutscher, cat#P04‐27005) with a 2% penicillin (10,000 U/ml) and streptomycin (10 mg/ml) solution (PS, Dutscher, cat#P06‐07100) and 1% bovine serum albumin (BSA, Dutscher, cat#P06‐1391100).

Each embryo was dispatched on numerating petri dish (35 mm of diameter). Tail of embryos was cut for genotyping (see 2.2 Animals section). Spinal cords were removed and pooled by genotype in ice‐cold medium of Leibovitz (L15) and treated for 20 min at 37°C with a trypsin–EDTA solution (Dutscher, cat#P10‐023100) at a final concentration of 0.05% trypsin and 0.02% EDTA. The dissociation was stopped by the addition of Dulbecco's modified Eagle's medium (DMEM) with 4.5 g/L of glucose (Dutscher, cat#P04‐03600), containing DNase I grade II (final concentration 0.5 mg/ml) (Dutscher, cat#P60‐37780100) and 10% fetal calf serum (FCS, Dutscher, cat#S15898S181B). Cells were mechanically dissociated by three forced passages through the tip of a 10‐ml pipette. Cells were then centrifuged at 180 x *g* for 10 min at +4°C on a layer of BSA (3.5%) in L15 medium. The supernatant was discarded, and the pellet was resuspended in a defined culture medium consisting of Neurobasal medium (Fisher scientific, cat#1894793) with a 2% solution of B27 supplement (Fisher scientific, cat#1899722), 2 mM of l‐glutamine (Dutscher, cat#3220316), 2% of PS solution (Fisher scientific, cat#4650416), and 10 ng/ml of brain‐derived neurotrophic factor (BDNF, Dutscher, cat#H151208). Viable cells were counted in a Neubauer cytometer, using the trypan blue exclusion test and were seeded at a density of 20 000 cells per well in 96‐well plates precoated with poly‐l‐lysine and were cultured at 37°C in a humidified air (95%)/CO_2_ (5%) atmosphere. Half of the medium was changed every other day.

Motoneurons were treated from days 1 to 9 with PXT864 (0.05 to 0.32 nM ACP and 12 to 80 nM BCL) alone or with riluzole (1 µM) to assess survival and maturity of SOD1^G93A^ motoneurons. To assess protective activity against glutamate intoxication, cells were exposed to PXT864 alone or with riluzole (1 µM) on day 13 for 1 hr before glutamate intoxication (5 µM for 20 min) and for a further 24 hr.

#### 2.4.3 Primary cortical neuron culture

Rat cortical neurons were cultured as described by Chumakov et al. (2014) and Callizot, Combes, Steinschneider, and Poindron (2013). Briefly pregnant female rats of 15‐day gestation were euthanized by cervical dislocation and the embryos were removed from the uterus. The cortex was removed and placed in ice‐cold medium of Leibovitz (L15; Pan Biotech, cat#P04‐27055) containing 2% of penicillin 10,000 U/ml and streptomycin 10 mg/ml (PS; Pan Biotech, cat#P06‐07100) and 1% of bovine serum albumin (BSA; Pan Biotech, cat#P06‐1391100). Cortex was dissociated by trypsin (0.05%, Pan Biotech, cat#P10‐023100) for 20 min at 37°C. The reaction was stopped by the addition of Dulbecco's modified Eagle's medium (DMEM; Pan Biotech, cat#P04‐03600) containing DNase I grade II (0.5 mg/ml; Pan Biotech, cat#P60‐37780100) and 10% of fetal calf serum (FCS; Invitrogen, cat#10270). Cells were then mechanically dissociated by three passages through a 10‐ml pipette. Cells were centrifuged at 515 g for 10 min at +4°C. The supernatant was discarded and pellet of cells was resuspended in a defined culture medium consisting of Neurobasal (Gibco, cat#21103) supplemented with B27 (2%; Gibco, cat#17504), l‐glutamine (2 mM; Pan Biotech, cat#P04‐80100), 2% of PS solution, and 10 ng/ml of brain‐derived neurotrophic factor (BDNF, Pan Biotech, cat#CB‐1115002). Viable cells were counted in a Neubauer cytometer using the trypan blue exclusion test. The cells were seeded at a density of 30,000 cells/well in 96‐well plates precoated with poly‐l‐lysine (Greiner, cat#655930) and were cultured at 37°C in a humidified air (95%)/CO_2_ (5%) atmosphere. After 12 days of culture, drugs were solved in culture medium (+0.1% DMSO) and then preincubated with neurons for 1 hr before glutamate injury. One hour after drug incubation, glutamate was added for 20 min, to a final concentration of 40 μM diluted in the presence of drugs. At the end of the incubation, medium was changed with medium with compounds and without glutamate. The culture was fixed 24 hr after glutamate injury.

### 2.5 *In vitro* Immunostaining and quantification

#### 2.5.1 NMJ and neurite network analysis in neuromuscular cocultures

**TABLE 2:** Description and list of antibodies

Antibody	Supplier	cat#	Species	RRID	Immunogen	Dilution
anti‐neurofilament 200 kDa antibody	Sigma‐Aldrich	N0142	Mouse, monoclonal	AB_477257	C‐terminal segment of enzymatically dephosphorylated pig neurofilament 200	1/400
anti‐microtubule‐associated protein 2 (MAP‐2) antibody	Sigma‐Aldrich	M4403	Mouse, monoclonal	AB_477193	rat brain microtubule‐associated proteins (MAPs)	1/400
anti‐neurofilament 200kDa antibody	Sigma‐Aldrich	N4142	Rabbit polyclonal	AB_477272	Neurofilament 200 from bovine spinal cord	1/1,000
anti‐TDP‐43 antibody	Cell Signaling	3448S	Rabbit polyclonal	AB_2271509	Synthetic peptide corresponding to residues surrounding Gly400 of human TDP43	1/100
anti‐rabbit Alexa Fluor 568	Invitrogen	A‐11011	Goat polyclonal	AB_143157	Rabbit immunoglobulins	1/400
anti‐mouse Alexa Fluor 488	Invitrogen	A32723	Goat polyclonal	AB_2633275	Mouse immunoglobulins	1/400

After treatment, NMJ were stained by incubating cells with α‐bungarotoxin coupled with Alexa 488 (500 nM, Molecular Probes, Cat# B13422) for 15 min at 37°C. Cells were then fixed with 4% paraformaldehyde (Sigma‐Aldrich) and axons were detected using a mouse monoclonal anti‐neurofilament 200 kDa antibody (1:400, Sigma‐Aldrich, Cat# N0142, RRID:AB_477257, Table 2), revealed with anti‐mouse Alexa Fluor 568 secondary antibody (1:400, Invitrogen, Cat#A11011, RRID:AB_143157). Images were acquired and analyzed using an InCell Analyzer™ 1000 (GE Healthcare) or ImageXpress (Molecular Devices) software.

#### 2.5.2 Motoneuron neurite network, maturity, and TDP‐43 analysis

Cells were fixed in a cold solution of 95% ethanol and 5% acetic acid. Neuron cell bodies and neurite networks were detected using mouse monoclonal anti‐microtubule‐associated protein 2 (MAP2) antibody (1:400, Sigma‐Aldrich, Cat# M4403, RRID:AB_477193). Neurons with a cell body diameter ≥15 µm and a minimum of three neuritic processes were considered as motoneurons (Bilsland, Nirmalananthan, Yip, Greensmith, & Duchen 2008). Motoneuron maturity was assessed by quantifying rabbit polyclonal anti‐neurofilament 200 kDa phosphorylated antibody (1:1,000, Sigma‐Aldrich, Cat# N4142, RRID:AB_477272) staining. TDP‐43 signal in cytoplasm was detected by rabbit polyclonal anti‐TDP‐43 antibody (1:100, Cell signaling, Cat# 3448S, RRID:AB_2271509). Primary antibodies were revealed with appropriate anti‐rabbit Alexa Fluor 568 (1:400, Invitrogen, cat#A‐11011, RRID:AB_143157) or anti‐mouse Alexa Fluor 488 (1:400, Invitrogen, cat#A32723, RRID:AB_2633275) secondary antibodies. Images were automatically acquired using ImageXpress (Molecular Devices). Motoneuron survival, maturity, neurite network, and TDP‐43 localization were analyzed automatically using Custom Module Editor software (Molecular Devices).

#### 2.5.3 Cortical neuron survival evaluation

After permeabilization with saponin (Sigma), cells were blocked for 2 hr with PBS containing 10% goat serum. Then cells were fixed, and neurites detected by using mouse monoclonal anti‐neurofilament antibody (Sigma‐Aldrich, Cat# N4142, RRID:AB_477272) revealed by an anti‐mouse Alexa Fluor 488 (1:400, Invitrogen, cat#A32723, RRID:AB_2633275). Total neurite length was analyzed using In Cell Analyzer™ 1000 (GE Healthcare).

### 2.6 TDP‐43 stress granules assay

This experiment was performed at Innoprot (Spain). A U2OS (human osteosarcoma) cell line stably overexpressing turbo‐GFP‐tagged human TDP‐43 was used to screen the effects of ACP (0.01 nM to 50 µM) and BCL (2 nM to 50 µM), alone or mixed in 36 PXT864 combinations, on TDP‐43 stress granule formation induced by sodium arsenate (Sigma‐Aldrich, cat#35000 Fluka) at 250 µM for 90 min. Briefly, recombinant TDP‐43‐tGFP‐U2OS cell line was thawed (2x10^6^ cells per T75) and were maintained in DMEM‐F12 supplemented with 10% FBS at 37°C in a humidified 5% CO_2_ atmosphere for 5 days. Cells were then plated in Poly‐l‐Lysine coated 96‐well plates (10,000 cells per well) and maintained in DMEM‐F12 medium supplemented with 10% FBS and 5 mM IPTG for 24 hr at 37°C in a humidified 5% CO_2_ atmosphere to induce TDP‐43‐tGFP expression. Drugs were applied to cultures 24 hr before arsenate addition. Pathological TDP‐43 stress granule formation was assessed prior to formaldehyde fixation (3.7%). Nuclei were stained using 2 µg/ml DAPI and fluorescence was measured using a BD Pathway 855 High‐Content Bioimager (Becton Dickinson). Numbers of TDP‐43‐GFP granules per cell were quantified using Attovision software (Becton Dickinson).

### 2.7 Statistical analysis

All analyses were performed using Prism 6 and 8.1 (Graphpad) and R version 3.4.3 (http://cran.r‐project.org). Statistical tests were two‐tailed and conducted at a 5% significance level. Analysis of Variance (ANOVA) with Dunnett's test was performed when comparing more than two groups against a reference. Additionally, *t*‐tests were used as a supportive technique.

Two effect‐based approaches of drug combination analysis were used to assess the synergy of treatment effects: the Bliss independence model and when not applicable, the Response Additivity analysis (Foucquier & Guedj, 2015) (Two‐way ANOVA, *α* = 5%). Among synergic combinations, only combinations exhibiting an effect significantly higher than the vehicle effect (Dunnett's test, *α* = 5%) were considered.

Neuron‐muscle coculture data were derived from five independent experiments with six replicates per experiment. Primary motoneuron data were derived from one experiment with six to 18 replicates. TDP‐43 stress granule assay data were derived from three independent experiments with three replicates. Results were normalized to the nontreated group (100%) or to the nontreated group (100%) versus glutamate or sodium arsenate‐intoxicated cells (0%) when applicable and expressed as min to max box plots or mean ± *SD* (Figures 1 and 5).

Cortical neuron primary cultures were repeated in three independent cultures with six replicates per condition.

## 3 RESULTS

### 3.1 Acamprosate and Baclofen act synergistically to protect neuron‐muscle cocultures against glutamate

Defective synaptic transmission at NMJs and glutamate‐induced excitotoxicity are common features of ALS (Battaglia & Bruno, 2018; King et al., 2016; Rocha et al., 2013). Therefore, protective potential of ACP and BCL was assessed in neuron‐muscle cocultures injured by the application of high dosage of glutamate. NMJ number and area were used as the indexes of muscle innervation level and quality, respectively, whereas neurite length was assessed for neuronal protection. Glutamate‐intoxicated cocultures were treated with a range of concentrations of ACP and BCL (Figure 1a) for which both drugs generally exhibited a bell‐shaped dose‐dependent protective activity on NMJ area and number, as well as on neurite length (Figure 1b–g). The protective potential of 25 combinations of ACP and BCL (PXT864) was then assessed. Ten combinations showed a significant protective activity on NMJ area (Figure 1h), six on NMJ number (Figure 1i) and nine on neurite length (Figure 1j). ACP and BCL acted synergistically in most of these active combinations, as assessed by the Bliss independence model, where the association of the two drugs showed greater protective effects than each drug alone. For instance, PXT864 containing 0.14 nM ACP and 80 nM BCL exhibited a substantial significant protective effect on all endpoints (73% and 63% on NMJ area and number, respectively, and 43% on neurite length), whereas single drugs did not show any significant effect (arrows and green bars in Figure 1k–m). Importantly, the effect of PXT864 was at least equal (Figure 1m) or significantly higher than the maximal effect of each drug alone (Figure 1k,l).

### 3.2 Combination of PXT864 and riluzole is not antagonistic in protecting against glutamate excitotoxicity

Continuous application of riluzole (Figure 2a) protected neuron‐muscle cocultures from glutamate excitotoxicity, as demonstrated by greater NMJ area (Figure 2b), number (Figure 2c), and neurite length (Figure 2d). Interestingly, when inactive or active PXT864 dosage was added to riluzole, their combination had greater protective effects than either alone (Figure 2b–e), highlighting the absence of antagonism but rather a positive interaction between the drugs.

### 3.3 PXT864 improves SOD1^G93A^ motoneuron survival and maturity

**FIGURE 3:**
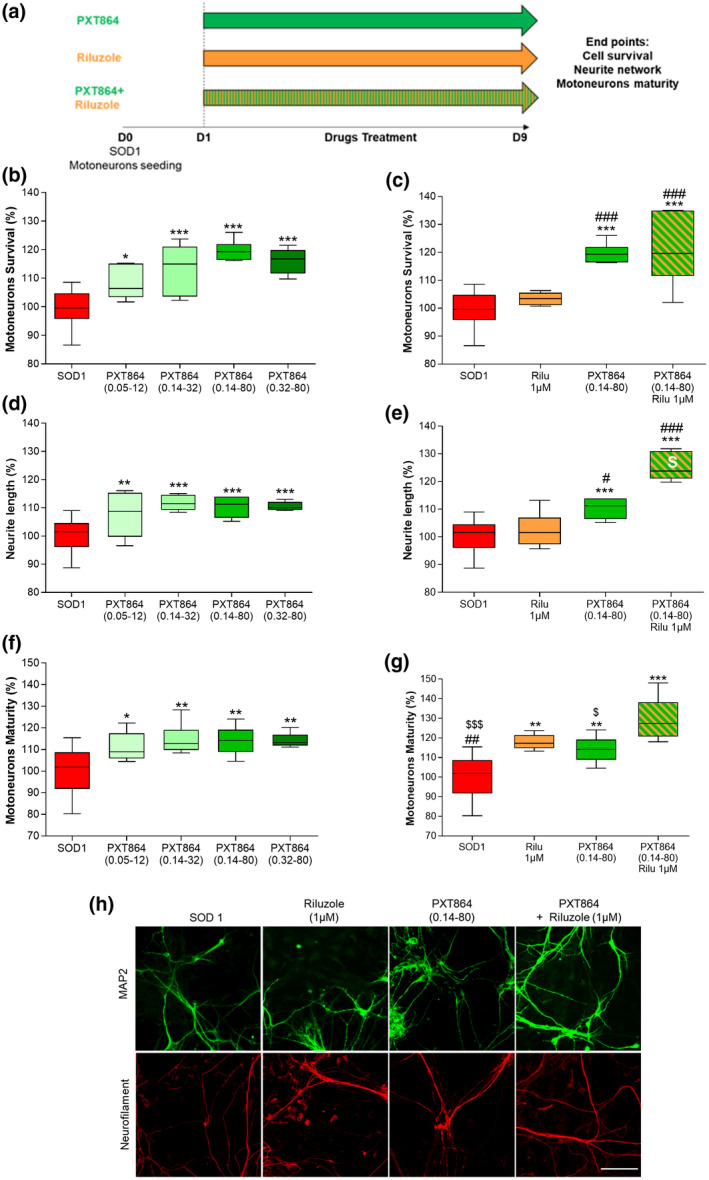
PXT864 improves SOD1^G93A^ motoneurons survival and maturity. (a) Schematic of SOD1^G93A^ motoneuron treatments. WT and SOD1^G93A^ embryos (E14) were used to generate primary culture of motoneurons (only SOD1 genotype was cultured). PXT864 protected SOD1^G93A^ motoneurons as assessed by MAP2‐positive neuron count (b) and MAP2‐positive neurite length (d). PXT864 also improved SOD1^G93A^ motoneuron maturity, assessed by the length of neurofilament (200 kDa) positive neurites (f). PXT864 (ACP 0.14 nM and BCL 80 nM) and riluzole acted synergistically to improve neurite length as assessed by response additivity analysis (white S symbol) (e) but not motoneurons survival (c). PXT864 (ACP 0.14 nM and BCL 80 nM) and riluzole combination had a higher protective activity on SOD1^G93A^ motoneuron maturity than each compound alone (g). In control condition (SOD1 without any treatment), value of 100 is equivalent to 48 ± 0.6 (b‐d‐f) and 47 ± 0.8 (c‐e‐g) motoneurons (per field). Representative pictures of the culture are presented in h, scale bar = 100 µm. Data are expressed as percentage of untreated SOD1 motoneurons and are presented as min to max boxplots. *p < 0.05; **p < 0.01; ***p < 0.001; treatment versus SOD1 control and ^#^ *p* < 0.05; ^##^ *p* < 0.01; ^###^ *p* < 0.001; treatment versus riluzole, ^$^ *p* < 0.05; ^$$$^ *p* < 0.001 treatment versus PXT864 + riluzole; assessed by ANOVA with Dunnett's test (b: *F*(4, 41) = 19.95, *p* < 0.0001, c: *F*(3, 37) = 25.84, *p* < 0.0001, d: *F*(4, 41) = 11.47, *p* < 0.0001, e: *F*(3, 37) = 39.11, *p* < 0.0001, f: *F*(4, 40) = 7.161, *p* = 0.0002, g: *F*(3, 35) = 18.74, *p* < 0.0001). Primary motoneuron culture data were derived from one experiment with six to 18 replicates [Color figure can be viewed at wileyonlinelibrary.com]

We then sought to assess PXT864 protective properties in another model of ALS, primary cultures of motoneurons derived from SOD1^G93A^ transgenic rat embryos. When ALS motoneurons were exposed to PXT864, riluzole, or both (Figure 3a), PXT864 not only improved motoneuron survival in a dose‐dependent manner (Figure 3b), but also increased neurite length (Figure 3d) and enhanced maturity defects observed in ALS motoneurons measured by neurofilament 200 kDa immunodetection (Figure 3f). Globally, the activity of PXT864 was superior (Figure 3c–e) or at least equal to riluzole (Figure 3g). Interestingly, PXT864 combination with riluzole had a greater protective potential on neurite length (Figure 3e) and motoneurons maturity (Figure 3g) than either PXT864 or riluzole alone, emphasizing again the positive interaction between PXT864 and the standard of care.

### 3.4 PXT864 protects SOD1^G93A^ motoneurons from glutamate‐induced excitotoxicity

**FIGURE 4:**
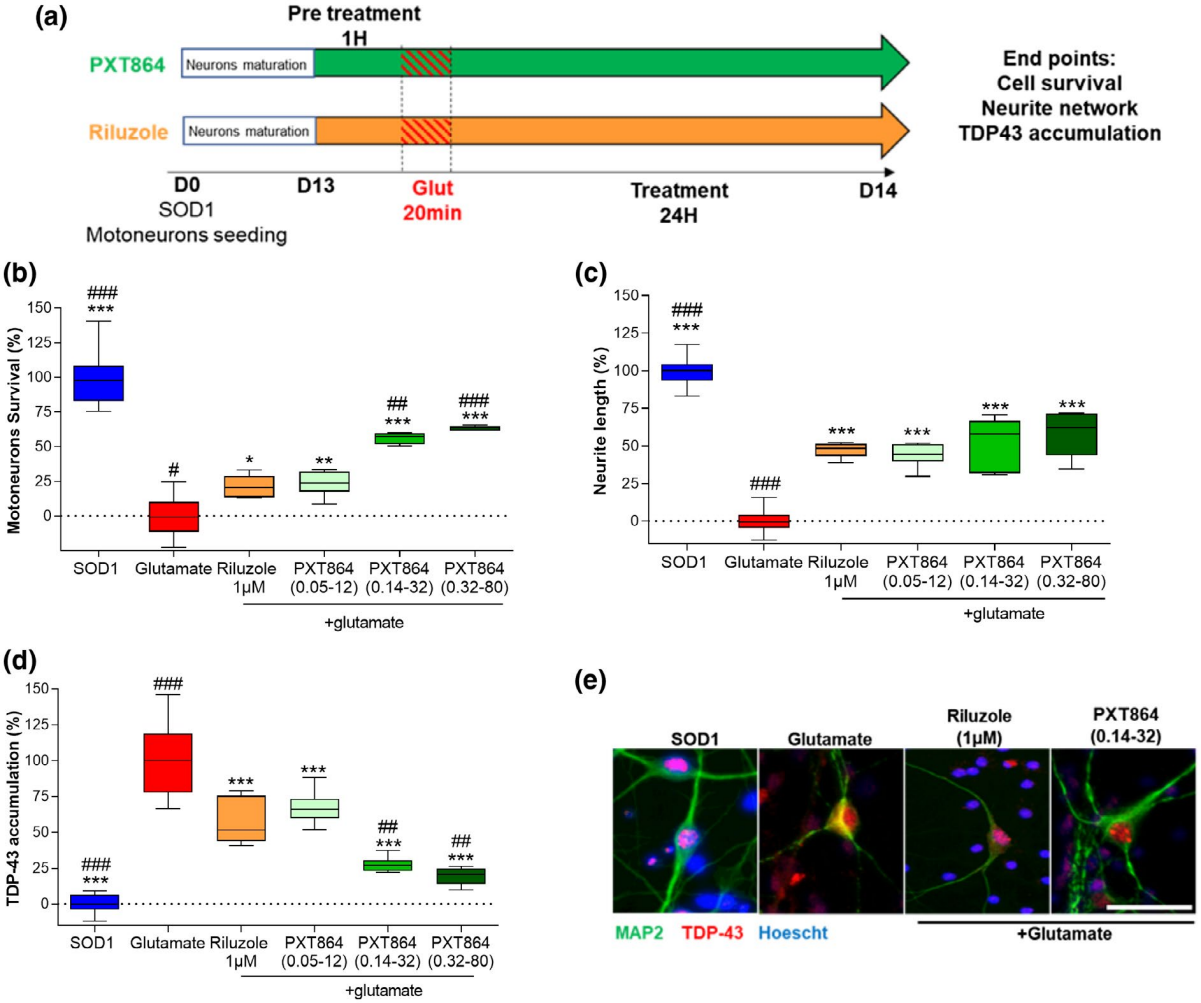
PXT864 protects SOD1^G93A^ motoneurons from glutamate toxicity. (a) Schematic of SOD1^G93A^ motoneuron treatments and glutamate intoxication. SOD1^G93A^ embryos (E14) were used to generate primary culture of motoneurons. PXT864 prevented SOD1^G93A^ motoneuron death (b) and neurite degeneration (c) in a dose‐dependent manner and to a greater extent than riluzole, as assessed by MAP2 immunostaining. TDP‐43 cytoplasmic accumulation in SOD1 motoneurons was measured by immunofluorescence (d) as illustrated in (e). PXT864 largely prevented TDP‐43 cytoplasmic accumulation induced by glutamate intoxication, to a greater extent than riluzole (d). In control condition (SOD1 without any treatment), value of 100 is equivalent to 45 ± 1.1 motoneurons (per field). Data are presented as min to max boxplots. *p < 0.05, **p < 0.01; ***p < 0.001 treatment versus glutamate condition and ^#^ *p* < 0.05, ^##^ *p* < 0.01; ^###^ *p* < 0.001 versus riluzole condition, assessed by one‐way ANOVA with Dunnett's test (b: *F*(5,49) = 96.07, *p* < 0.0001; c: *F*(5, 51) = 154.9, *p* < 0.0001; d: *F*(5,46) = 77.72, *p* < 0.0001). Scale bar = 50 µm (e). Primary motoneuron cultures data were derived from one experiment with six to 18 replicates [Color figure can be viewed at wileyonlinelibrary.com]

As glutamate‐induced excitotoxicity contributes to ALS pathogenesis, SOD1^G93A^ motoneurons were challenged by glutamate (Figure 4a). PXT864 protected SOD1^G93A^ motoneurons from glutamate toxicity in a dose‐dependent manner and had a significant greater effect on motoneuron survival than riluzole (Figure 4b). PXT864 was also significantly effective on the protection of neurite length (Figure 4c).

TDP‐43 leakage from the nucleus and accumulation in cytoplasmic granules is a hallmark of ALS (Neumann et al., 2006). Glutamate stress‐induced TDP‐43 cytoplasmic accumulation in motoneurons was counteracted by PXT864 dose dependently with a significant greater activity than riluzole, reaching non‐intoxicated vehicle levels (Figure 4d,e).

### 3.5 PXT864 prevents TDP‐43 stress granule formation

**FIGURE 5:**
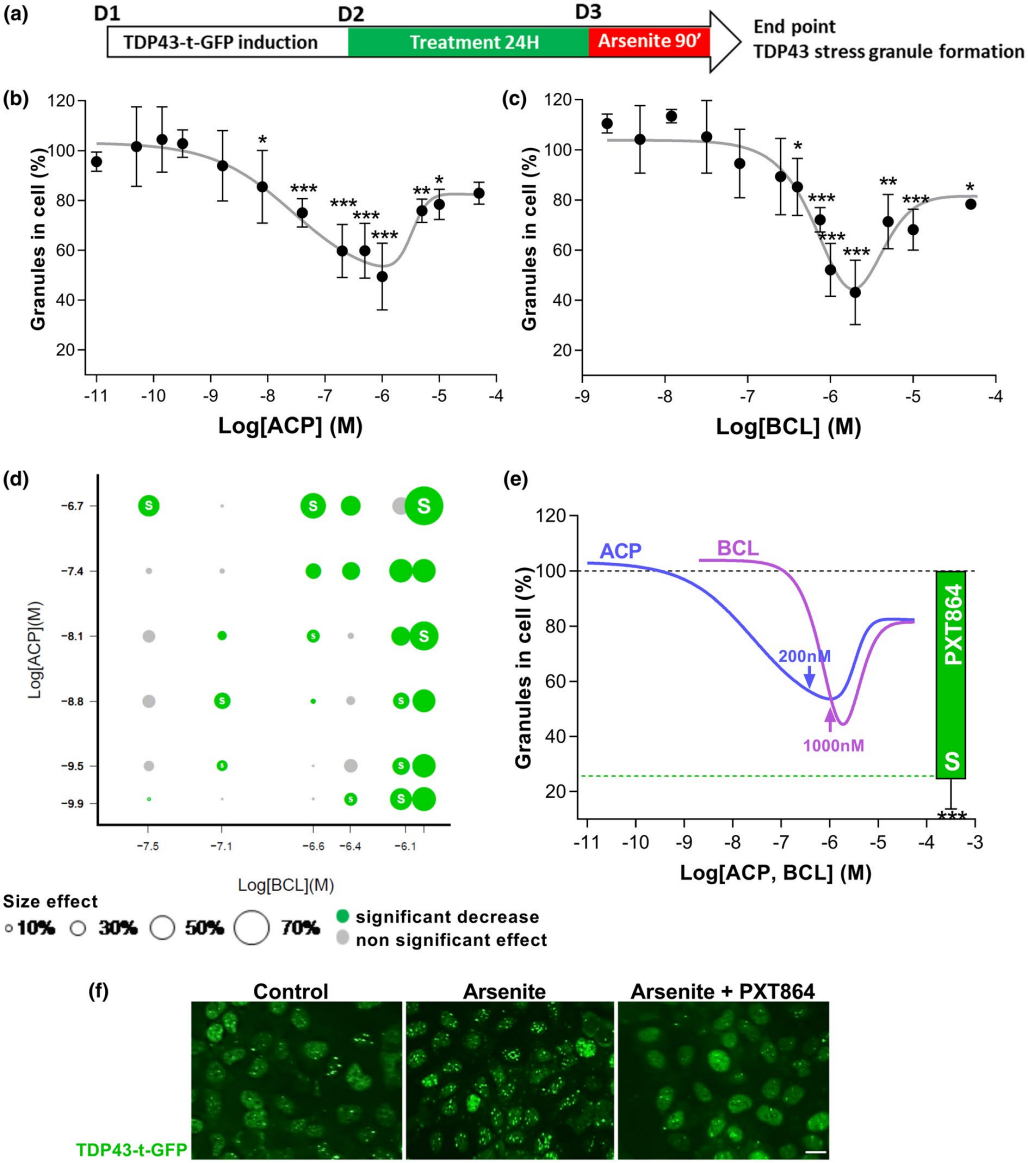
PXT864 prevents TDP‐43 stress granules formation. (a) Schematic of treatment of U2OS cell line overexpressing human TDP‐43 fused to turbo‐GFP prior to sodium arsenate (250 µM) induction of stress granule formation. BCL (2 nM to 50 µM) and ACP (0.01 nM to 50 µM) inhibited stress granule formation in a dose‐dependent manner (b and c). Of 36 PXT864 combinations tested, 23 significantly inhibited stress granule formation and among these 11 were synergistic as determined by Bliss independence model (white S) (d). PXT864 (ACP 200 nM and BCL 1,000 nM) showed higher activity than maximal activity of each single drug (e). Effect of PXT864 (ACP 200 nM + BCL 1,000 nM) on stress granule is illustrated in (f). Data are presented as mean ± *SD*. *p < 0.05, **p < 0.01; ***p < 0.001; treatment versus arsenate condition, assessed by ANOVA with Dunnett's test (b: *F*(14, 75) = 56.10, *p* < 0.0001; c: *F*(14, 75) = 55.61, *p* < 0.0001). Scale bar = 20 µm (f). TDP‐43 stress granule assay data were derived from three independent experiments with three replicates per experiment (Total: *n* = 9) [Color figure can be viewed at wileyonlinelibrary.com]

Under various cellular stresses, such as oxidative stress, cytosolic structures composed of assembled riboproteins such as TDP‐43 are formed to stop protein translation. To assess PXT864 effect on stress granule formation, U2OS cells overexpressing TDP‐43 fused to turbo‐GFP were treated with ACP, BCL, or PXT864 before stress granule formation upon induction of oxidative stress by arsenate (Figure 5a). ACP and BCL prevented TDP‐43 stress granule formation in a U‐shaped dose‐dependent manner (Figure 5b,c). Then, ACP (0.14 nM to 200 nM) and BCL (32 nM to 1,000 nM) were mixed in 36 PXT864 combinations. Among these, 63% were significantly active and 30% acted synergistically on TDP‐43 stress granule formation, as assessed by the Bliss independence model (Figure 5d). The greatest effect was achieved by PXT864 containing 200 nM ACP and 1,000 nM BCL with an inhibitory activity of 76% that was superior to the maximal effects of ACP or BCL when tested individually (Figure 5e,f). In this model, ACP and BCL proved again their importance to be combined in PXT864 for the achievement of high effectiveness at low doses.

### 3.6 PXT864 protects cortical neurons against glutamate induced excitotoxicity

**FIGURE 6:**
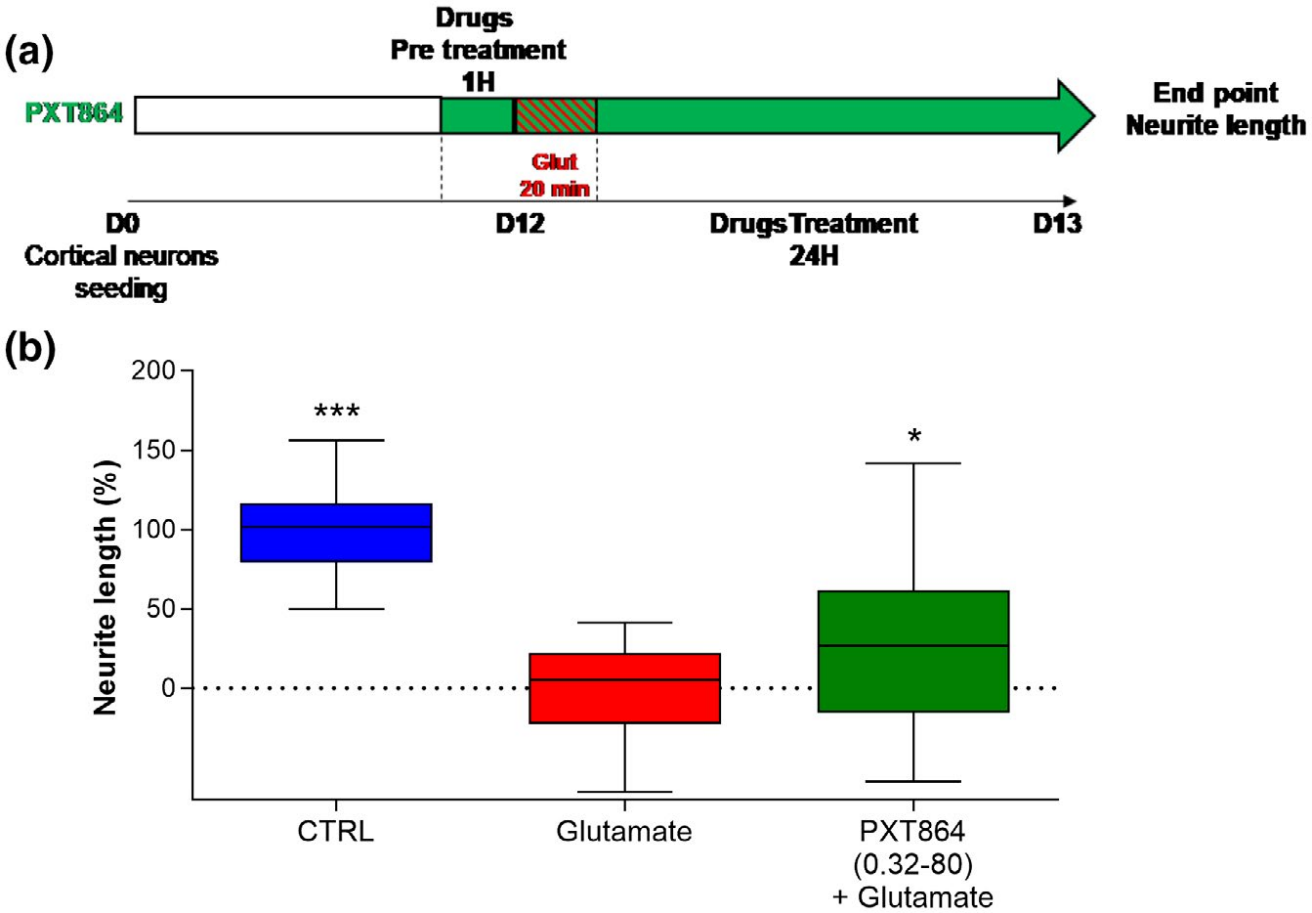
PXT864 protects cortical neurons from glutamate toxicity. (a) On day 12 of culture, rat cortical neurons were pretreated 1 hr with PXT864 (0.32 nM ACP, 80 nM BCL) before glutamate intoxication (20min). PXT864 was then incubated for additional 24 hr, and neurite length was assessed by neurofilament immunostaining. (b) PXT864 protects cortical neurons against glutamate toxicity. Data are presented as min to max boxplots. *p < 0.05; ***p < 0.001; treatment versus glutamate condition assessed by ANOVA with Dunnett's test (*F*(2, 105) = 80.88, *p* < 0.0001). Data were derived from three independent cultures with six replicates per condition (Total *n* = 18) [Color figure can be viewed at wileyonlinelibrary.com]

As ALS disease affects both upper and lower motoneurons, we also assessed PXT864 activity on primary cultures of cortical neurons (Figure 6). PXT864 (ACP 0.32 nM + BCL 80 nM) significantly protected cortical neurons from glutamate injury.

## 4 DISCUSSION

Overall PXT864, a combination of ACP and BCL, protected NMJ, motoneurons, and cortical neurons against glutamate‐induced excitotoxicity. Interestingly, PXT864 improved riluzole's protection and reduced TDP‐43 stress granule formation.

Although more than 50 drugs targeting various pathophysiological mechanisms of ALS have been studied, only two compounds have come to the market: riluzole and edaravone. Riluzole is a relatively safe drug and is speculated to reduce glutamatergic transmission (Cheah et al., 2010). However, its efficacy has turned to be poor and very short‐lasting (Lacomblez et al., 1996). Edaravone is a free radical scavenger mediating elimination of lipid peroxides and hydroxyl radicals by an unknown mechanism. The efficacy of this drug is very limited as well, and was only assessed in a highly selected subset of ALS patients (Group. WGE (MCI‐186) A 19 S, 2017). Thus, there is still an unmet medical need for alternative treatments for ALS (Fang et al., 2018).

Excitotoxicity is a complex mechanism involving the dysregulation of extracellular glutamate concentration and overactivation of NMDA receptors resulting in calcium overload and neuronal death (King et al., 2016). Increased glutamate release along with reduced glutamate uptake by astrocytes were widely described as direct excitotoxicity mechanisms (Do‐Ha et al., 2018). Other important observations point to the impaired regulation of motoneuron firing by interneurons in both ALS patients and animal models. Interneurons regulate motoneuron activity by releasing inhibitory neurotransmitters such as GABA and glycine in the spinal cord (Jonas, Bischofberger, & Sandkühler, 1998). Cortical and spinal cord interneurons are both affected in ALS (Ince et al., 1993; Stephens et al., 2006). GABA inhibition is described to be reduced in ALS patient's cortex, as shown by TMS measurement, and correlates with disease duration and severity (Zanette et al., 2002a, 2002b). In ALS patients, spinal cord glycine concentration is also reduced (Malessa, Leigh, Bertel, Sluga, & Hornykiewicz, 1991) and its receptor (GLRA1) expression is decreased in a murine ALS model (Chang & Martin, 2011). Overall, these elements point to the imbalance between excitatory and inhibitory transmission (Kiernan, Ziemann, & Eisen, 2019) contributing in part to motoneuron death. In this context, we adopted a multitargeting strategy combining ACP and BCL (PXT864), two well‐known drugs with favorable safety profiles. ACP is currently used for relapse prevention in alcoholism and is thought to attenuate the hyper‐glutamatergic state observed in early abstinence (Dahchour et al., 1998). Its precise mechanism of action is still unknown, but it is thought to modulate glutamate receptors and inhibitory glycine‐gated ion channels. BCL is a GABA_B_ agonist currently used to treat spasticity in ALS (Yoon et al., 2017). Importantly, PXT864 efficacy was already demonstrated in AD and PD models (Chumakov et al., 2015; Hajj et al., 2015) which share molecular and cellular pathogenic mechanisms with ALS, particularly glutamatergic and GABAergic imbalance. Based on these rational observations, we assessed PXT864 activity in models of ALS.

Promoting both motoneuron axonal and NMJ integrity has been shown to delay muscle atrophy and improve muscle performance in SOD1^G93A^ mice (Seijffers et al., 2014). Communication between motoneurons and muscles is bidirectional and axonal “dying back” degeneration is well described in human ALS and murine models (Dadon‐Nachum et al., 2011; Fischer et al., 2004; Rocha et al., 2013). Maintaining axonal connectivity with the muscle and promoting motoneuron survival are both essential to stabilize or prevent ALS progression. These reasons prompted us to use a coculture of neurons and muscle cells as a model of ALS primarily assessing NMJ and motoneuron neurite integrity. Using this model, we demonstrated that PXT864 protected both motoneuron neurite length and NMJ integrity. Importantly, BCL and ACP acted synergistically against glutamate injury, and their combination PXT864 had greater effects than the maximal effect of either drug alone. Interestingly, PXT864 also interacted positively with riluzole to the improve protection of neuron‐muscle cocultures against excitotoxicity, which paves the way of testing PXT864 clinically in patients already treated with riluzole.

The initial demonstration of PXT864 positive activity in the neurons‐muscle coculture model was further supported in another cellular model of ALS in which purified motoneurons were derived from spinal cords of SOD1^G93A^ rat embryos. In the absence of glutamatergic stress, PXT864 improved motoneuron survival, neurite network, and axon maturity. The protective effect of PXT864 was stronger when glutamate was used as toxic agent, emphasizing a greater positive action of the combination when experimental conditions were closer to the physiopathology of the disease. In line with previous data from the coculture model, the effect of PXT864 in combination with riluzole was also found to be advantageous, confirming the potential future use of PXT864 with the standard of care as a promising investigational treatment in ALS patients. The mechanism of action underlying the positive interaction between PXT864 and riluzole is not yet understood. Future studies could allow elucidating this synergistic effect. Wild‐type cultures were excluded from this work on SOD1 motoneurons as we preferred to restrict our assessments to disease conditions for which any improvement with such a drug combination could be meaningful to patients, regardless of the size of the effect achieved by the treatment. We were also guided by clinical assessments under which only patients are randomized and tested for investigational drugs without any comparison to normal subjects.

Motoneuron cultures used in this study contained approximately 10%–20% of inhibitory interneurons. Besides the direct action on motoneurons, one other possible mechanism of PXT864 might be its direct action on interneurons to improve the inhibition of motoneurons by reducing their hyperexcitable state, which should be addressed in future studies. As upper cortical neurons are also affected in ALS inducing in turn the neurodegeneration of lower motoneurons (de Carvalho, Eisen, Krieger, & Swash, 2014), another hypothesis could consist in protecting upper neurons with PXT864 to improve lower motoneuron activity. We verified in part this hypothesis by testing the effect of PXT864 on cortical neurons and found the protective effect of the combination against glutamate‐induced excitotoxicity (Figure 6). Similar observations were also previously made on the protective ability of PXT864 from oxidative stress (Chumakov et al., 2015).

Overall, *in vitro* results demonstrated the positive activity of PXT864 in ALS. The combination acted synergistically *in vitro* whether ACP and BCL were combined each at low inactive doses, or at higher doses close to their maximal effect. These results pointed also to a mechanism of action potentially involving the regulation of protein misfolding and clearance. PXT864 prevented TDP‐43′s cytoplasmic accumulation in motoneurons and stress granule formation *in vitro*. TDP‐43 intracellular inclusions have been consistently reported in both sporadic and familial ALS (Bosco et al., 2010; Neumann et al., 2006). The propensity of mutant proteins to misfold and form aggregates is a common factor defining neurodegenerative diseases. Strategies aiming in part to rescue abnormal protein clearance could afford protection against motoneuron degeneration and extend survival in animal models of ALS (Medinas, Valenzuela, & Hetz, 2018; Saxena, Cabuy, & Caroni, 2009). Therefore, on the one hand, it would be of interest to determine whether PXT864 action on protein aggregates could be mediated by indirect or direct modulation of protein clearance pathways. It is well established that glutamate toxicity induces calcium overload that leads to both proteasome‐ubiquitin system downregulation and to caspase, calpains, ER stress, and autophagy activation (Ayala et al., 2011; Berning & Walker, 2019; Caldeira et al., 2013; Crippa et al., 2016; Djakovic, Schwarz, Barylko, DeMartino, & Patrick 2009; Yap et al., 2016), disturbing protein processing and degradation pathways. The decreasing of glutamate/GABA imbalance by PXT864 may indirectly restore protein homeostasis and misfolded protein clearance (Caldeira et al., 2013; Djakovic et al., 2009) leading to an improvement in disease conditions. Moreover, direct involvement of target proteins modulated by PXT864 in regulation of protein folding/clearance is also not excluded. For example, GABA_B_ receptors have been shown to bind directly to CHOP and ATF4 proteins, two key transcription factors implicated in control of ER stress (Lim & Yue, 2015; Yap et al., 2016); as well, recent publications demonstrated that GABA_B_ receptors are able to suppress cyto‐destructive autophagy and downregulate the activity of PERK, ATF4, and CHOP proteins in degenerating neurons under hypoxic conditions (Fu, Wu, Hu, Li, & Gao, 2016; Kim et al., 2018; Liu et al., 2015; Nehring et al., 2000; Sauter et al., 2005). Further investigations of PXT864 effects on neuronal excitation/inhibition balance and on cellular pathways involved in protein clearance, as well as on the regulation of SOD1 and TDP‐43 expression, will allow a deeper understanding of its mechanisms of action.

## 5 CONCLUSIONS

In the present study, we brought evidence on the ability of PXT864 to protect NMJs and motoneurons from glutamate excitotoxicity. Such effects could be partly explained by the capacity of PXT864 to prevent TDP‐43 accumulation and stress granule formation. Last, PXT864 interacted positively with riluzole paving the way toward its use in addition on top of the standard of care in patients suffering from this life‐threatening disease. The converging evidence from different cellular models relevant for the disease strengthen the therapeutic potential of such a safe combination to be tested in animal models of ALS or directly in patients due to the lack of robust animal models of this devastating disease and the urgent need of new therapeutic options.

These results highlight the importance of combining ACP and BCL to achieve a positive efficacy and demonstrate the potential value of combining polytropic‐acting drugs as a promising new strategy to treat ALS.

## DECLARATION OF TRANSPARENCY

The authors, reviewers and editors affirm that in accordance to the policies set by the *Journal of Neuroscience Research*, this manuscript presents an accurate and transparent account of the study being reported and that all critical details describing the methods and results are present.

## ETHICS APPROVAL

Our manuscript reports animal experimentation that was approved by local ethics committees.

## CONFLICT OF INTEREST

All the authors except Noëlle Callizot are employee of Pharnext. RH, SN, and DC are cited in patents held by Pharnext. The authors declare this study was founded by Pharnext. The funder had the following involvement with the study: study design, data collection and analysis, decision to publish and preparation of the manuscript.

## AUTHOR CONTRIBUTIONS

All mentioned authors contributed to this work. *Conceptualization*: D.C. and R.H. *Methodology*: R.H. and L.B. *Candidate drugs*: S.N. *Validation*: R.H., L.B., N.C. *Formal Analysis*: J.L., P.S., P.R. *Investigation:* R.H., L.B., N.C. *Writing‐original draft*: L.B. and R.H. *Writing ‐ Review & editing*: L.B., R.H., J.L., P.S., P.R., S.N., N.C., D.C. *Visualization*: L.B., R.H. and J.L. *Supervision*: R.H. and D.C. *Project administration*: R.H.

### PEER REVIEW

The peer review history for this article is available at https://publons.com/publon/10.1002/jnr.24714.

## Supporting information

Transparent Peer Review Report

Transparent Science Questionnaire for Authors

## Data Availability

Data supporting these findings are available from the corresponding author upon request.
