# Supplementary material for: Combination of acamprosate and baclofen (PXT864) as a potential new therapy for amyotrophic lateral sclerosis
Source: J Neurosci Res. 2020 Aug 19;98(12):2435–50. doi: 10.1002/jnr.24714 (PMC7693228; doi:10.1002/jnr.24714)
Supplement: Supplementary file 1 — Transparent Peer Review Report [file JNR-98-2435-s001.docx]

**Combination of acamprosate and baclofen (PXT864) as a potential new therapy for amyotrophic lateral sclerosis**

Boussicault, Lydie (contact); Laffaire, Julien; Schmitt, Peter; Rinaudo, Philippe; Callizot, Noëlle; Nabirotchkin, Serguei; Hajj, Rodolphe; Cohen, Daniel

_____________________________________________________________________________________

| Review timeline: | Submission date: 12-Feb-2020  Editorial Decision: Conditional Reject (RER) (25-Apr-2020)  Revision Received: 02-Jun-2020  Editorial Decision: Minor Modification (12-Jul-2020)  Revision Received: 16-Jul-2020  Accepted: 25-Jul-2020 |  |
| --- | --- | --- |
|  |  |  |

Editor: Howland, Dena

Reviewer 1:	Borchelt, David

Reviewer 2:	Lin, Glenn

_____________________________________________________________________________________

**1st Editorial Decision	25-Apr-2020**	____________________________________________________________________________________

Dear Dr Boussicault:

Thank you for submitting your manuscript to the Journal of Neuroscience Research. We've now received the reviewer feedback and have appended those reviews below. As you will see, the reviewers find the question addressed to be of potential interest. Yet, they do not find the manuscript suitable for publication in its current form, have identified a series of problems and indicated that considerable further work would be necessary to support your conclusions.

If you feel that you can adequately address the concerns of the reviewers, you may revise and resubmit your paper within 90 days. It will require further review. Please explain in your cover letter how you have changed the present version. If you require longer than 90 days to make the revisions, please contact Dr Cristina Ghiani (cghiani@mednet.ucla.edu). You can submit your revised manuscript directly by clicking on the following link: *** PLEASE NOTE: This is a two-step process. After clicking on the link, you will be directed to a webpage to confirm. ***

https://mc.manuscriptcentral.com/jnr?URL_MASK=2cde39238569427994f628d698f58f9d

Thank you again for your submission to the Journal of Neuroscience Research; we look forward to reading your revised manuscript.

Best Wishes,

Dr Dena Howland
Associate Editor, Journal of Neuroscience Research

Dr Cristina Ghiani
Co-Editor-in-Chief, Journal of Neuroscience Research


Associate Editor: Howland, Dena
Comments to the Author:
Based upon the collective reviews, the submitted manuscript requires substantial revision before it can be reconsidered for publication.  Both reviewers highlighted technical concerns, lack of clear, detailed methodology, a need for pharmacological data and dosing rationale, difficulty with figures, and a need for additional experiments and/or analyses to support the major conclusions of the study.  If the issues raised in each review are considered and addressed when revising the manuscript, the journal would be happy to consider a re-submission.

Additional Editorial Comments:
1. The total number of animals used must be reported.

2. Were the female Wistar and SOD1G93A rats purchased and used for in vitro experiments pregnant? if so, the pregnancy time should be stated. Also it should be mentioned for what in vitro exp they were used, how they were euthanized.

3. Cell culture, please briefly describe.

4. Figure legends should be more clear, it must be stated at the beginning whether the data shown were obtained in mice, primary cultures (mice or rats?) cell lines. In addition, the number of animals/assay should be added.

5. JNR does not support the usage of bar graphs, please be sure all continuous data plots are depicted as scatterplots or box and whisker plots rather than bar graphs to better visualize the distribution of data. In addition, Pleas be sure to review the following, to ensure accurate graphical visualization and transparent reporting: https://goo.gl/w5dnYa and https://onlinelibrary.wiley.com/doi/epdf/10.1002/jnr.24340
STATISTICAL TESTS
Articles containing statistical analyses should state the name of the statistical test, the n value for each statistical analysis, the comparisons of interest, and justification for the use of the test. It should be clear what statistical test was used to generate every P value. Moreover, the authors must include the values from the appropriate statistical test (e.g., F(x,x) = xx; n = x; P = x.xxx). If the tests violate any assumptions, the authors must provide this information.


6. GRAPHICAL ABSTRACT
Please upload a graphical abstract, which we are asking of all authors submitting original research articles. This is intended to provide readers with a visual representation of the conclusions and an additional way to access the contents and appreciate the main message of the work. What we require is a .tif image file and a .doc text file containing an abbreviated abstract. For the image, labels, although useful, must be kept to a minimum and the image should be 400 x 300, 300 x 400, or 400 x 400 pixels square and at a resolution of 72 dpi. This can be one of the figures from your article, or something slightly different, as long as it represents your study.  Instructions for this can be found in our author guidelines online at http://onlinelibrary.wiley.com/journal/10.1002/(ISSN)1097-4547/homepage/ForAuthors.html

7. please move the SIGNIFICANCE STATEMENT after the abstract.

8. RESOURCE IDENTIFICATION INITIATIVE (Abbreviated Text)
Please incorporate Research Resource Identifiers (RRIDs) in your citation of all resources used in your manuscript (antibodies, software tools, databases, model organisms) where applicable in the text, exactly as you would a regular citation or Genbank Accession number.  Please also be sure these RRIDs are included in your keywords list in addition to the required keywords. For any antibodies, we ask that you also include the RRIDs in your antibody table, in addition to citing them in the text. An example of how to list RRIDs in your antibody table can found in the example antibody table attached and for more information about how to obtain and cite RRIDs within your text, please visit the "Resource Identification Initiative" section of our author guidelines: http://onlinelibrary.wiley.com/journal/10.1002/(ISSN)1097-4547/homepage/ForAuthors.html

Furthermore, in accord with JNR requirements, you will have to provide additional information on each of the antibodies that you use.  This must include the source (not just the manufacturer and catalog or lot number, but the species it was raised in, and the EXACT structure of the immunizing antigen - including the amino acid sequence for peptide antigens); characterization (e.g., what does it recognize on immunoblots); and appropriate controls (i.e. the effect of blocking peptides for immunohistotochemical localization).  

Reviewer: 1

Comments to the Author
The manuscript by Boussicault describes a pre-clinical assessment of the potential utility of a novel combination of 2 drugs for ALS that are currently in use in humans in other conditions.  The study was funded and conducted by a for-profit entity called Pharnext.  Appropriate statements of conflict are present. It looks like most of the work was performed by third party providers, but it would be useful to provide a table that summarizes the origins of data for the various experiments. There is quite a bit of quantitative data in the paper, and I strongly recommend that excel files with the raw quantitative data be provided as supplementary data.  It would also be useful, it possible, to obtain a statement by the companies that performed the work that the data provided in the paper are accurately reported and complete.
The paper contains evidence that low dose combinations of accmprosate and baclofen (designated PXT8640) shows protection against glutamate toxicity in two different model systems.  The drugs also showed neuroprotection in primary motor neuron cultures derived from G93A SOD1 mice. Most of these data are straightforward with a few minor issues (listed below).  The in vivo studies in the G93A mouse were modestly encouraging, and here is would be very helpful to have some pharmacological data on the levels of the drugs in the CNS of the treated mice.  It would also have been very useful to have assessed whether the drug treatments preserved NMJ’s in the mice.  

1) In the experiments involving primary G93A rat motor neurons, it is not clear whether the cultures were mixtures of WT and G93A cells.  The cultures were prepared from E14 embryos.  Was each embryo individually cultured?  If not, then what was the mixture of WT and G93A cells in the culture?  On page 12, line 245, it is stated that the primary motor neuron data were derived from 1 experiment with 6 to 18 replicates.   Although I would prefer to see multiple experiments, it might be acceptable to have one experiment with multiple replicate if the replicates are individually cultured embryos.  
2) The methodology used in assessing TDP43 cytoplasmic accumulation is unclear (Fig. 4d-e). How many independent cultures were examined?  How many cells per culture?  Issues raised above also apply here.
3) The location of the objects that are presumed to be stress granules is a bit unusual.  These do not appear to be cytoplasmic.  I agree that arsenite has induced in increase in nuclear puncta, but these structures are not the same as cytoplasmic stress granules.  
4) The methodology used in assessing SOD1 a protein aggregation in Fig 7b-d are uncler.  Are the small puncta in the bottom middle panel the aggregates?  How many sections per animal were quantified?  What part of the spinal cord was examined? The version of this figure that I received is missing labels.  
5) It would be helpful to the reader if the first paragraph of the Discussion summarized the main findings.


Reviewer: 2

Comments to the Author
In the current manuscript, the authors provide evidence that (1) submaximal combination doses of ACP and BCL (PXT864) provide protective, synergistic effects greater that either compound alone in 2 different SOD1-G93A in vitro models, (2) PXT864 combinatorial effects may be further synergized with additional treatment of the approved drug riluzole, (3) PXT864 may provide this protection by reduction of excitotoxicity and may reduce TDP-43 stress granule formation, and (4) PXT864 may provide mild to modest delays in disease progression of the SOD1-G93A mouse model as assessed by behavioral analysis and histology. While this manuscript may provide insight into the therapeutic potential of PXT864 in ALS, the following concerns remain.
(1) Overall for the animal study, this reviewer have significant concerns about the statistical validity of the reported data. While it is appropriate to separate male and female data for analysis, further subdividing the sexes further reduces the statistical validity of the experiments (n = 8M/12F). Furthermore, the survival data (with n = 4 – 7 per sex per group) is even less statistically reliable. In general, the current number of animals reported is too low for a behavioral or survival study.    
(2) This reviewer has concerns about the treatment timing in the animal study. Treatment began at p63. This represents a pre-symptomatic stage in SOD1-G93A mice. The age p80-90 is considered the early symptomatic stage and a more clinically-relevant time-point to start treatment to assess efficacy of a compound in this animal model. Why was this time point for treatment not chosen or not followed up with after the initial proof of principle, pilot study?
(3) The authors consistently observed benefits of PXT864 (0.14 nM ACP & 80 nM BCL) in both in vitro models. How was the in vivo dosing selected? Is the lumbar spinal cord (or at least the brain) Cmax of each compound at the dose treated in vivo similar to the in vitro models? This especially seems pertinent as the higher doses of ACP and BCL actually reduced survival/protection of motor neurons in in vitro studies.
(4) What is the rationale for 2 doses per day instead of 1? Is this based on the half-life and the proposed mechanism of action of the drug? If so, this pharmacokinetic data regarding the compound should be shown or at least discussed and referenced.
(5) In the SOD1-G93A mice, only lumbar motor neuron survival was analyzed for histopathology. It would seem to be reasonable to also analyze if PXT864 has a similar neuroprotective effect at the neuromuscular junction in vivo as was seen in the in vitro studies.
(6) One of the major ideas espoused throughout this article was the idea that PXT864 would be considered a combinational therapy with Riluzole. Why is there not a Riluzole alone and at least on Riluzole + PXT864 group in the SOD1-G93A in vivo study?
(7) Figure 2E appears to be missing 2 panels of representative images that should be incorporated.
(8) Figure 3 & 4 are missing representative images for motor neuron survival, neurite length, and maturity (Fig 3).
(9) In Figures 2 & 4, the panels that include “+ glutamate” are mislabeled as the riluzole group is not underlined suggesting it was not treated with glutamate. This was quite confusing and should be addressed.
(10) Figure 7 is a disaster. It is possible that it did not upload correctly initially. Therefore, it is almost impossible to interpret the data presented for Fig 7.
(11) Figure 3 is lacking a control (WT) group. This group should be incorporated because it is important to see how much restoration PXT864 provides for each parameter assessed.
(12) For Fig 6 H & I, it is inappropriate to report mean survival for SOD1-G93A mice and should be reported as median and assessed statistically by Kaplan Meier survival analysis.

_____________________________________________________________________________________

**Authors’ Response	02-Jun-2020**

The authors are very grateful to the editors and reviewers for their careful reading of the manuscript, their comments and helpful advices. The authors modified the manuscript according to the editor’s recommendations; please find below our detailed answers and comments.
In order to fulfill the reviewers concerns on animal data, the authors decided to remove the in vivo section from the manuscript. The authors agree indeed with the reviewers’ opinion but would nevertheless like to precise that these data are derived from a pilot study with a small sample size of males and females. We agree indeed that more animals are needed to achieve a better conclusion on the efficacy of the drug combination in animals, but the data is however still promising. Unfortunately, the authors cannot initiate immediately a new study aiming to improve the number of animals, the sex ratio, the dosage, treatment duration or read out (including pharmacological study). In consequence, the authors propose to re-submit their work without in vivo data. We removed all supplementary material and included in the main manuscript data obtained in primary culture of cortical neurons (new figure 6).

Additional Editorial comment:
1) The total number of animals used must be reported.
Total number of animals used for in vivo study was 97 (n=20; 8 males and 12 females per genotype except for the higher dose: n=17; 7 males and 10 females per genotype)”. This part is now removed from the manuscript.

2) Were the female Wistar and SOD1G93A rats purchased and used for in vitro experiments pregnant? if so, the pregnancy time should be stated. Also it should be mentioned for what in vitro exp they were used, how they were euthanized.
Wistar and SOD1G93A female rat were pregnant, this information is now added in material and method section with detailed time of pregnancy: “For in vitro experiments, pregnant female Wistar (pregnancy time = E13) and SOD1G93A rats (pregnancy time = E14) were purchased from Janvier Labs (France) and Taconic bioscience (USA) respectively” (p6, line 94).

WT Wistar female rats were used for neuron/muscle co-culture while SOD1G93A pregnant rat were used to generate motoneuron cocultures, this information is now added in the appropriate material and method section.

We thank the editor to highlight the lack of precision on rat euthanasia, this is now added to material and method section: “Animals were euthanized using a deep anesthesia with CO2 chamber and a cervical dislocation” (p6, line 97).

3) Cell culture, please briefly describe.
Following editor recommendations, authors added details on neuron/muscle co-culture, motoneurons and cell line cultures method in the material and method section (p6, line 109; p8, line 139 and p11, line 225 respectively).

4) Figure legends should be more clear, it must be stated at the beginning whether the data shown were obtained in mice, primary cultures (mice or rats?) cell lines. In addition, the number of animals/assay should be added.
The authors thank the editor for this comment and followed this recommendation by adding in the legends more details about experimental condition.

5) JNR does not support the usage of bar graphs, please be sure all continuous data plots are depicted as scatterplots or box and whisker plots rather than bar graphs to better visualize the distribution of data. In addition, Please be sure to review the following, to ensure accurate graphical visualization and transparent reporting: https://goo.gl/w5dnYa and https://onlinelibrary.wiley.com/doi/epdf/10.1002/jnr.24340
The authors modified all graph formats to fit with JNR recommendation by presenting when possible min to max boxplots.

6) GRAPHICAL ABSTRACT
Please upload a graphical abstract, which we are asking of all authors submitting original research articles. This is intended to provide readers with a visual representation of the conclusions and an additional way to access the contents and appreciate the main message of the work. What we require is a .tif image file and a .doc text file containing an abbreviated abstract. For the image, labels, although useful, must be kept to a minimum and the image should be 400 x 300, 300 x 400, or 400 x 400 pixels square and at a resolution of 72 dpi. This can be one of the figures from your article, or something slightly different, as long as it represents your study. Instructions for this can be found in our author guidelines online at http://onlinelibrary.wiley.com/journal/10.1002/(ISSN)1097-4547/homepage/ForAuthors.html
The authors generated a graphical abstract that is now available for the journal.

7) please move the SIGNIFICANCE STATEMENT after the abstract.
The authors moved the significance statement after the abstract.

8) STATISTICAL TESTS
Articles containing statistical analyses should state the name of the statistical test, the n value for each statistical analysis, the comparisons of interest, and justification for the use of the test. It should be clear what statistical test was used to generate every P value. Moreover, the authors must include the values from the appropriate statistical test (e.g., F(x,x) = xx; n = x; P = x.xxx). If the tests violate any assumptions, the authors must provide this information.
Statistics: The authors included the values of the statistical tests in the legend of each figure.

9) RESOURCE IDENTIFICATION INITIATIVE (Abbreviated Text) Please incorporate Research Resource Identifiers (RRIDs) in your citation of all resources used in your manuscript (antibodies, software tools, databases, model organisms) where applicable in the text, exactly as you would a regular citation or Genbank Accession number. Please also be sure these RRIDs are included in your keywords list in addition to the required keywords. For any antibodies, we ask that you also include the RRIDs in your antibody table, in addition to citing them in the text. An example of how to list RRIDs in your antibody table can found in the example antibody table attached and for more information about how to obtain and cite RRIDs within your text, please visit the "Resource Identification Initiative" section of our author guidelines: http://onlinelibrary.wiley.com/journal/10.1002/(ISSN)10974547/homepage/ForAuthors.html
The authors added RRID when appropriate and a table describing antibodies and additional information needed in material and method section.

_____________________________________________________________________________________


Reviewer: 1

Comments to the Author
The manuscript by Boussicault describes a pre-clinical assessment of the potential utility of a novel combination of 2 drugs for ALS that are currently in use in humans in other conditions. The study was funded and conducted by a for-profit entity called Pharnext. Appropriate statements of conflict are present. It looks like most of the work was performed by third party providers, but it would be useful to provide a table that summarizes the origins of data for the various experiments. There is quite a bit of quantitative data in the paper, and I strongly recommend that excel files with the raw quantitative data be provided as supplementary data. It would also be useful, if possible, to obtain a statement by the companies that performed the work that the data provided in the paper are accurately reported and complete.
The paper contains evidence that low dose combinations of acamprosate and baclofen (designated PXT864) shows protection against glutamate toxicity in two different model systems. The drugs also showed neuroprotection in primary motor neuron cultures derived from G93A SOD1 mice. Most of these data are straightforward with a few minor issues (listed below). The in vivo studies in the G93A mouse were modestly encouraging, and here is would be very helpful to have some pharmacological data on the levels of the drugs in the CNS of the treated mice. It would also have been very useful to have assessed whether the drug treatments preserved NMJ’s in the mice.

The authors are very grateful to reviewer 1 for its careful reading of the manuscript and his/her comments and suggestions.
Please find below a table summarizing for each figure the origins of the data. If necessary, we can include this table as supplementary material.

Figures Model Origin of the data
1 Nerve/muscle co-culture Neuronexpert (France) and Neuro-Sys (France)
2 Nerve/muscle co-culture Neuronexpert (France) and Neuro-Sys (France)
3 Primary motoneuron culture (SOD1G93A rat) Neuro-Sys (France)
4 Primary motoneuron culture (SOD1G93A rat) Neuro-Sys (France)
5 U2OS expressing turbo GFP-TDP43 cell line Innoprot (Spain)
6 Primary cortical neurons Neuronexpert (France)
Table summarizing the origin of the data

Raw data files generated by our providers will be available upon request as precise in availability statement section as JNR does not require systematic publication of raw data in supplementary material. The work presented in this paper results from collaborative analysis of the results between Pharnext and its providers scientists. It is noteworthy that one of the co-authors of this publication is the scientific leader of a provider that performed most of the in-vitro data.
The authors agree with the reviewer that in vivo study is modestly encouraging. This study should be considered as a pilot study paving the way to further study aiming to improve combination dosage, to include new parameters such as NMJ’s and collection of pharmacological data and to confirm PXT864 efficacy.
For these reasons, the authors removed in vivo data and propose to the reviewer a new version of the paper containing only in vitro data.

However, the authors would like to present some pharmacological data they obtained on SOD1G93A mice. BCL and ACP levels were measured in mice plasma by using a HPLC system and MS/MS detection. ACP detection needs further optimization so the authors cannot include this results in the publication.


Baclofen and acamprosate are detected in plasma. Baclofen and acamprosate concentrations were measured in treated mice plasma samples collected 2 hours after treatment administration (dose 1: ACP 0.2mg/kg+ BCL 3mg/kg; dose 2: ACP 0.5mg/kg+ BCL 7.5mg/kg, dose 3: ACP 1mg/kg+ BCL 15mg/kg) (a and b) (n=8 mice per group, statistics: + p<0.05 student t test; *** p<0.001 ANOVA one-way vs dose 1 followed by Dunnett’s test (a: F(2, 20)= 87.08, p<0.001, b: F(2, 16)=2.630, p=0.1029, t test p=0.04)).

1) In the experiments involving primary G93A rat motor neurons, it is not clear whether the cultures were mixtures of WT and G93A cells. The cultures were prepared from E14 embryos. Was each embryo individually cultured? If not, then what was the mixture of WT and G93A cells in the culture? On page 12, line 245, it is stated that the primary motor neuron data were derived from 1 experiment with 6 to 18 replicates. Although I would prefer to see multiple experiments, it might be acceptable to have one experiment with multiple replicate if the replicates are individually cultured embryos.
Each embryo was dispatched on numerating petri dish (35 mm of diameter). Tail of embryos was cut for genotyping and after genotyping determination, embryos were pooled by genotype. WT embryos were excluded and only SOD1 embryos were seeded. Indeed, the experiments were performed on pure SOD1 motoneuron population. This precision is now added to the material and method section (p8, Line 143). The embryos were not cultured individually as material collected from 1 embryo was not sufficient to perform enough assay with replicates.

2) The methodology used in assessing TDP43 cytoplasmic accumulation is unclear (Fig. 4d-e). How many independent cultures were examined? How many cells per culture? Issues raised above also apply here.
In this experiment one culture with 6 replicates were performed. As precised in the precedent point, cultures resulted from a pool of SOD1 motoneurons. More detailed culture protocol is now available in Material and Methods section (p8, line 139). TDP43 was detected by immunofluorescence and automatic quantification of TDP43 area signal outside the nucleus as we observed that TDP43 localization is restricted to nucleus in normal condition. The data represents TDP43 accumulation in cytoplasm. Analysis was performed on 900 to 1400 neurons (30 to 45 motoneurons per field x 30 fields).

3) The location of the objects that are presumed to be stress granules is a bit unusual. These do not appear to be cytoplasmic. I agree that arsenite has induced in increase in nuclear puncta, but these structures are not the same as cytoplasmic stress granules.
The granule localization and morphology correspond to the published behavior shown by TDP43 after arsenite treatment (or other toxic agents). As an example, reviewer 1 can find stress granule illustration in the manuscript published by Mann JR et al., Cell, 2019 (https://doi.org/10.1016/j.neuron.2019.01.048), in particular in figure 5. Perhaps the low magnification level of the pictures is misleading but, the phenotype observed in our experiment is similar and repeated in other models.

4) The methodology used in assessing SOD1 a protein aggregation in Fig 7b-d are unclear. Are the small puncta in the bottom middle panel the aggregates? How many sections per animal were quantified? What part of the spinal cord was examined? The version of this figure that I received is missing labels.
In this figure the authors measured SOD1 staining area and intensity, but they did not quantify SOD1 aggregation as at 16 weeks-old these structures were hard to detect in SOD1 mice. Data were collected in lumbar part of the spinal cord using 2 section arrays containing 2 lumbar sections each (n=8 animals). This figure is now removed from the paper.

5) It would be helpful to the reader if the first paragraph of the Discussion summarized the main findings.
The authors added a summary of principal findings at the beginning of the discussion section (p16, line 325): “Overall PXT864, a combination of acamprosate and baclofen, protected both NMJ, motoneurons and cortical neurons against glutamate induced excitotoxicity. Interestingly, PXT864 improved riluzole protection. PXT864 also reduced TDP43 granule stress formation.”


Reviewer: 2

Comments to the Author
In the current manuscript, the authors provide evidence that (1) submaximal combination doses of ACP and BCL (PXT864) provide protective, synergistic effects greater that either compound alone in 2 different SOD1-G93A in vitro models, (2) PXT864 combinatorial effects may be further synergized with additional treatment of the approved drug riluzole, (3) PXT864 may provide this protection by reduction of excitotoxicity and may reduce TDP-43 stress granule formation, and (4) PXT864 may provide mild to modest delays in disease progression of the SOD1-G93A mouse model as assessed by behavioral analysis and histology. While this manuscript may provide insight into the therapeutic potential of PXT864 in ALS, the following concerns remain.

(1) Overall for the animal study, this reviewer have significant concerns about the statistical validity of the reported data. While it is appropriate to separate male and female data for analysis, further subdividing the sexes further reduces the statistical validity of the experiments (n = 8M/12F). Furthermore, the survival data (with n = 4 – 7 per sex per group) is even less statistically reliable. In general, the current number of animals reported is too low for a behavioral or survival study.
The authors agree that the low number of mice may have a negative impact on the statistical analysis to prove the effect of PXT864. However, these results are encouraging such a positive significant effect was shown with this number of mice. The authors agree that a further study will be required to confirm this effect or improve it with different doses of PXT864 (ratio change) in this or another model of ALS. The small sample size used in the study is due to gender differences already described in the discussion. Larger sample size is indeed needed in future study as initially discussed.
This in vivo study should be considered as pilot study, in consequence, the authors propose a new version of the manuscript without in vivo data. We modified the take home message of our work to highlight the potential of PXT864 in ALS that should be assessed in in vivo models or tested directly in patients due to the safe nature of the combination. (See conclusion section, p20, line 431).

(2) This reviewer has concerns about the treatment timing in the animal study. Treatment began at p63. This represents a pre-symptomatic stage in SOD1-G93A mice. The age p80-90 is considered the early symptomatic stage and a more clinically-relevant time-point to start treatment to assess efficacy of a compound in this animal model. Why was this time point for treatment not chosen or not followed up with after the initial proof of principle, pilot study?
The authors agree with the reviewer that treatment initiation and drug dosage should be improved in a further study. We would like to highlight that this study was the pilot study and despite some weaknesses, encouraging results were obtained. This time point was chosen based on literature and internal experience with this model. In this particular study, the authors and their collaborators could observe a delay in symptoms appearance that they could not explain.

(3) The authors consistently observed benefits of PXT864 (0.14 nM ACP & 80 nM BCL) in both in vitro models. How was the in vivo dosing selected? Is the lumbar spinal cord (or at least the brain) Cmax of each compound at the dose treated in vivo similar to the in vitro models? This especially seems pertinent as the higher doses of ACP and BCL actually reduced survival/protection of motor neurons in in vitro studies.
In vivo dosage was chosen based on previous efficacy experiment performed on Alzheimer and Parkinson mice model (Hajj et al., 2015; Chumakov et al., 2014 and unpublished results). Correlation between in vitro and in vivo model is difficult to make because of important differences in drugs absorption and distribution. The authors did not measure ACP and BCL level in mice spinal cord but this experiment should be included in a further study.

(4) What is the rationale for 2 doses per day instead of 1? Is this based on the half-life and the proposed mechanism of action of the drug? If so, this pharmacokinetic data regarding the compound should be shown or at least discussed and referenced.
The rationale for 2 doses is based on previous experiment performed in Alzheimer and Parkinson mice model. Pharmacokinetics experiments were performed in WT mice and showed that half-life of the drugs were short (around 5 hours) and therefore treatment necessitated a twice a day oral administration.

(5) In the SOD1-G93A mice, only lumbar motor neuron survival was analyzed for histopathology. It would seem to be reasonable to also analyze if PXT864 has a similar neuroprotective effect at the neuromuscular junction in vivo as was seen in the in vitro studies.
The authors agree with the reviewer, such parameter should be assessed in a further study.

(6) One of the major ideas espoused throughout this article was the idea that PXT864 would be considered a combinational therapy with Riluzole. Why is there not a Riluzole alone and at least on Riluzole + PXT864 group in the SOD1-G93A in vivo study?
This in vivo study was a pilot study and already involved 100 animals because 3 doses of PXT864 were assessed. For ethical reasons and because riluzole effect in SOD1G93A mice is controversial, the author decided to focus their study on PXT864. Once the optimal dose of PXT864 defined in this mice model, studies aiming to combine PXT864 and riluzole should be addressed.

(7) Figure 2E appears to be missing 2 panels of representative images that should be incorporated.
This figure contains numerous panels and for sake of clarity, the authors chose to only illustrate PXT864 (ACP 0.14nM + BCL 80nM) as this combination is representative of both combinations presented in this figure. To avoid reduction of the pictures that are already small, the authors prefer to not add supplementary pictures.

(8) Figure 3 & 4 are missing representative images for motor neuron survival, neurite length, and maturity (Fig 3).
Pictures are now added to illustrate MAP2 and Neurofilament staining.

(9) In Figures 2 & 4, the panels that include “+ glutamate” are mislabeled as the riluzole group is not underlined suggesting it was not treated with glutamate. This was quite confusing and should be addressed.
The authors thank the reviewer for his/her comment and corrected figure 2 and 4 accordingly.

(10) Figure 7 is a disaster. It is possible that it did not upload correctly initially. Therefore, it is almost impossible to interpret the data presented for Fig 7.
The authors apologized for the quality of the figure and removed this figure and in vivo results from the publication.

(11) Figure 3 is lacking a control (WT) group. This group should be incorporated because it is important to see how much restoration PXT864 provides for each parameter assessed.
We would like to thank the reviewer for this relevant comment.
Wildtype cultures were excluded from this work on SOD1 motoneurons as we preferred to restrict our assessments to disease conditions for which any improvement with such a drug combination could be meaningful to patients, regardless of the size of the effect achieved by the treatment. We were also guided by clinical assessments under which only patients are randomized and tested for investigational drugs without any comparison to normal subjects. This statement has been now added to the discussion section (p18, line 382) for clarity on the lack of a WT condition.

(12) For Fig 6 H & I, it is inappropriate to report mean survival for SOD1-G93A mice and should be reported as median and assessed statistically by Kaplan Meier survival analysis.
In vivo data were removed from the manuscript.

_____________________________________________________________________________________

**2^nd^ Editorial Decision	12-Jul-2020**

Dear Dr Boussicault:

Thank you for resubmitting your manuscript to the Journal of Neuroscience Research. We've now received the reviewer feedback and have appended those reviews below.  All reviews are very positive and only minor revisions are requested.  Please revise based upon points from each reviewer, including the addition of data sources to the methods section (information included in the rebuttal letter).  We look forward to receiving your revised manuscript.  

These points should be relatively straightforward to address. If there are any questions or points that are problematic, please feel free to contact me. I am glad to discuss.

We ask that you return your manuscript within 30 days. Please explain in your cover letter how you have changed the present version. If you require longer than 30 days to make the revisions, please contact Dr Cristina Ghiani (cghiani@mednet.ucla.edu). You can submit your revised manuscript directly by clicking on the following link: *** PLEASE NOTE: This is a two-step process. After clicking on the link, you will be directed to a webpage to confirm. ***

https://mc.manuscriptcentral.com/jnr?URL_MASK=24c13f77085b4dbd89a359eeeaf1935f

Thank you again for your submission to the Journal of Neuroscience Research; we look forward to reading your revised manuscript.

Best Wishes,

Dr Dena Howland
Associate Editor, Journal of Neuroscience Research

Dr Cristina Ghiani
Editor-in-Chief, Journal of Neuroscience Research


Associate Editor: Howland, Dena
Comments to the Author:
All reviews are very positive and only minor revisions are requested.  Please revise based upon points from each reviewer, including the addition of data sources to the methods section (information included in the rebuttal letter).  We look forward to receiving your revised manuscript.  

Statistics Editor: McArthur, David
Comments to the Author:
Use of the term "p<0.0015" is quizzical.  You have four digit decimal accuracy in all reported p-values and have used p<0.0001 appropriately.  Kindly change those "p<0.0015" entries to their correct values of <0.0001 if applicable.

The use of SEM in Figures 1 and 5 is not provided with a rationale ("SEM" was struck from the original ms in Section 2.9).  SD's are preferred.  SEMS's calculated on separate data distributions that all have identical means and identical variances will be the same only if the number of cases in all groups is also the same.  Thus SEMs cannot be interpreted as a stable reflection of variability for a given set of data distributions, and the visualizations using SEMs are thus also not stable.  It is for these reasons that 21st century statistical thinking has pushed for SDs.

The panel pairs within Figure 3 (b+c; d+e; f+g) map identical terms on the y-axis but the y-axis limits are not the same across pairs.  The triplet of panels in Figure 5 b+c+e also have identical y-axis terms but varying y-axis limits.  Consistency would be strongly preferred.  Also, error bars are missing from Figure 5e.  But then is Figure 5e simply repeating panels b and c?  If that is the case this set of panels needs to be reduced to a, d and e where e uses the dot\error bar\line strategy of b and c.

Minor points:  Figure 2 caption: "NMJ area (B)" needs to strike "(B)" and "NMJ number (per field) (C)" needs to strike "(C)". The "$" and "$$$" symbols used in Figure 3g are undefined.  Figure 4 caption: "greater extent than riluzole (b)" needs to strike the "(b)".  Figure 4 caption: "45 +/- 1.1 (b) motoneurons" needs to strike "(b)".  Figure 5 caption: "of each single drugs" needs "drug" singular.

Reviewer: 1

Comments to the Author
If acceptable with the journal, I would add the information in the table on origins of data (rebuttal letter) to the methods section - line 108. Otherwise, the revised manuscript adequately addresses my concerns.

Reviewer: 3

Comments to the Author
The manuscript by Boussicault et al., describes investigation of effect of PXT864 containing a combination of acamprosate and baclofen on parameters relevant to the pathophysiology of the ALS disease. The authors measure the effect of these two drugs separately and together and also in combination with an approved ALS drug, riluzole. The authors test these drugs on  neuron-muscle co-cultures subjected to glutamate toxicity which is relevant to ALS. Also, the authors test these compounds on the familial ALS mutant SOD-G93A bearing motoneuron’s survival. They also test the ability of these compounds on prevention of formation of TDP-43’s stress granules which is also a feature relevant to ALS. Authors show that the combination formulation, PXT864, is better at alleviating ALS-related neuronal damages than the individual components and that PXT864 can also synergistically boost riluzole towards these effects.
The data presented is clear, supportive of the conclusions drawn. The study is important considering the dearth of treatment options for ALS and the low effectiveness of the approved drugs. Although the authors have removed the data on the in vivo experiments on mice models from their previous version of the manuscript, the revised manuscript with the in vitro data is still supportive of the conclusions made so far by the authors regarding the potential applicability of PXT864 and the need for further investigations. Thus, I recommend the publication of this manuscript in JNR with the following minor corrections:

1. Line 170:  “days gestation were killed by cervical dislocation”, Change to “euthanized”.
2. Line 309: “before stress granule formation induction of oxidative”  add “upon” before “induction”.
3. Line 325: Delete “both”.
4. Line 326: Change “riluzole protection” to “riluzole’s protection”.
5. Line 326; Line 707, Line 714 & Figure 5a: Change “TDP43 granule stress” to “TDP43 stress granule”.
6. Line 402: Change “TDP-43 cytoplasmic” to “TDP-43’s cytoplasmic”.
7. Line 411: Change “CASPASE” to “caspase”.
8. Figure 5f: Indicate “TDP-43-tGFP” on the left side of the panel.


*********IMPORTANT: Instructions and checklists follow*********
When finalized, please upload your complete revised manuscript onto our website, preferably as a word document. Please ensure to upload a highlighted version of your manuscript along with the clean version. The highlighted version should highlight the revised text or any other changes made to the manuscript. The clean version should have no highlighted sentences, strike-through words, or comments in margins. Kindly avoid submitting a document with tracked changes. Figures must be uploaded separately in .tif or eps format. Please review our submission checklist, which can be found in our author guidelines and also be sure to fill out the Transparent Science Questionnaire attached to this email.

JNR offers Open Science badges to qualifying authors. For more information please see the “Open Science initiatives” section of our author guidelines. If you would like to apply for one or more of the badges, please complete the included disclosure form and upload it as Supplemental Material Not for Review when submitting your final manuscript files.

To submit your revised manuscript:
*You have two ways to submit your revision. Either click on the link: *** PLEASE NOTE: This is a two-step process. After clicking on the link, you will be directed to a webpage to confirm. ***

https://mc.manuscriptcentral.com/jnr?URL_MASK=0e41e828eceb47039b01f35d8286479b or Log on to https://mc.manuscriptcentral.com/jnr using your case-sensitive User ID ((Person not available)).

For security purposes your password is not listed in this email. If you are unsure of your password you may click the link to set a new password.
(Person not available)

*Enter the Author Center, where you will find your manuscript title listed under “Manuscripts with Decisions”
* Under “Actions,” click on “Create a Revision”. Your manuscript number has been appended to denote a revision.
*Follow the prompts and replace existing files with revised ones, as necessary.

If you encounter any troubles in submitting your revised manuscript, please contact Dr Cristina Ghiani (cghiani@mednet.ucla.edu) or click on Get Help Now at the top right of any ScholarOne Manuscripts screen.

_____________________________________________________________________________________

**Authors’ Response	16-Jul-2020**

Dear Editors,

The authors are very grateful to the editors and reviewers for their careful reading of the new version of the manuscript and theirs very positive comments. The authors modified the manuscript according to the editor’s and reviewer’s recommendations; please find below our detailed answers and comments.

Use of the term "p<0.0015" is quizzical. You have four digit decimal accuracy in all reported p-values and have used p<0.0001 appropriately. Kindly change those "p<0.0015" entries to their correct values of <0.0001 if applicable.
The authors thank the editor for highlighting this typographic error and change “p<0.0015” by p=0.0015 in the legend of figure 1 (p27, L654-655).

The use of SEM in Figures 1 and 5 is not provided with a rationale ("SEM" was struck from the original ms in Section 2.9). SD's are preferred. SEMS's calculated on separate data distributions that all have identical means and identical variances will be the same only if the number of cases in all groups is also the same. Thus SEMs cannot be interpreted as a stable reflection of variability for a given set of data distributions, and the visualizations using SEMs are thus also not stable. It is for these reasons that 21st century statistical thinking has pushed for SDs.
The authors replaced SEM error bars by SD’s in figure 1 and 5. This is now pointed in material and method section (p12, L252) and in legend of figures 1 (p27, L652) and 5 (p30, L715).

The panel pairs within Figure 3 (b+c; d+e; f+g) map identical terms on the y-axis but the y-axis limits are not the same across pairs. The triplet of panels in Figure 5 b+c+e also have identical y-axis terms but varying y-axis limits. Consistency would be strongly preferred. Also, error bars are missing from Figure 5e. But then is Figure 5e simply repeating panels b and c? If that is the case this set of panels needs to be reduced to a, d and e where e uses the dot\error bar\line strategy of b and c.
The authors are grateful to the editor for his comments.
Figure 3: the authors harmonized y-axis limits according to editor recommendation.
Figure 5: Y-axis limits was homogenized as requested by the editor. Panel e) was designed to show the superiority of PXT864 over its single compound. Dose response curves of BCL and ACP are quite similar, and the authors are afraid that merging the 2 graphs presented in b and c with dots and error bars will be confusing for the reader. As in figure 1, we prefer to present first the proof of single compound efficacy and then the proof of mix superiority for sake of clarity.

Minor points: Figure 2 caption: "NMJ area (B)" needs to strike "(B)" and "NMJ number (per field) (C)" needs to strike "(C)". This point is now corrected in figure caption (P28, L 667-668).

The "$" and "$$$" symbols used in Figure 3g are undefined. The authors are grateful to the editor for this comment and add the definition of these symbols in figure 3 caption (P29, L 688).

Figure 4 caption: "greater extent than riluzole (b)" needs to strike the "(b)". Figure 4 caption: "45 +/- 1.1 (b) motoneurons" needs to strike "(b)". Figure 5 caption: "of each single drugs" needs "drug" singular. These points are corrected in figure 4 and 5 captions (P29, L697 and 701 and P30, L714).

Reviewer: 1

Comments to the Author
If acceptable with the journal, I would add the information in the table on origins of data (rebuttal letter) to the methods section - line 108. Otherwise, the revised manuscript adequately addresses my concerns.
The author thank reviewer 1 for her/his recommendation and for her/his positive opinion on the revised manuscript. The table was added to the end of the manuscript (P26, L632) and introduce in methods section (P6, L107).

Reviewer: 3

Comments to the Author
The manuscript by Boussicault et al., describes investigation of effect of PXT864 containing a combination of acamprosate and baclofen on parameters relevant to the pathophysiology of the ALS disease. The authors measure the effect of these two drugs separately and together and also in combination with an approved ALS drug, riluzole. The authors test these drugs on neuron-muscle co-cultures subjected to glutamate toxicity which is relevant to ALS. Also, the authors test these compounds on the familial ALS mutant SOD-G93A bearing motoneuron’s survival. They also test the ability of these compounds on prevention of formation of TDP-43’s stress granules which is also a feature relevant to ALS. Authors show that the combination formulation, PXT864, is better at alleviating ALS-related neuronal damages than the individual components and that PXT864 can also synergistically boost riluzole towards these effects.
The data presented is clear, supportive of the conclusions drawn. The study is important considering the dearth of treatment options for ALS and the low effectiveness of the approved drugs. Although the authors have removed the data on the in vivo experiments on mice models from their previous version of the manuscript, the revised manuscript with the in vitro data is still supportive of the conclusions made so far by the authors regarding the potential applicability of PXT864 and the need for further investigations. Thus, I recommend the publication of this manuscript in JNR with the following minor corrections:

1. Line 170: “days gestation were killed by cervical dislocation”, Change to “euthanized”.
2. Line 309: “before stress granule formation induction of oxidative” add “upon” before “induction”.
3. Line 325: Delete “both”.
4. Line 326: Change “riluzole protection” to “riluzole’s protection”.
5. Line 326; Line 707, Line 714 & Figure 5a: Change “TDP43 granule stress” to “TDP43 stress granule”.
6. Line 402: Change “TDP-43 cytoplasmic” to “TDP-43’s cytoplasmic”.
7. Line 411: Change “CASPASE” to “caspase”.
8. Figure 5f: Indicate “TDP-43-tGFP” on the left side of the panel.

The authors are very grateful to reviewer 3 for her/his careful reading of the manuscript and her/his positive comments. All these corrections were applied following reviewer 3 recommendations.

_____________________________________________________________________________________

**3^rd^ Editorial Decision	25-Jul-2020**

Dear Dr Boussicault:

Thank you for submitting your manuscript "Combination of acamprosate and baclofen (PXT864) as a potential new therapy for amyotrophic lateral sclerosis" by Boussicault, Lydie; Laffaire, Julien; Schmitt, Peter; Rinaudo, Philippe; Callizot, Noëlle; Nabirotchkin, Serguei; Hajj, Rodolphe; Cohen, Daniel.

You will be pleased to know that We are pleased to accept your manuscript for publication in the Journal of Neuroscience Research.

In the coming weeks, the Production Department will contact you regarding a copyright transfer agreement and they will then send an electronic proof file of your article to you for your review and approval.

Please note that your article cannot be published until the publisher has received the appropriate signed license agreement. Within the next few days, the corresponding author will receive an email from Wiley’s Author Services asking them to log in. There, they will be presented with the appropriate license for completion. Additional information can be found at https://authorservices.wiley.com/author-resources/Journal-Authors/licensing-open-access/index.html

Congratulations on your results, and thank you for choosing the Journal of Neuroscience Research for publishing your work. I hope you will consider us for the publication of your future manuscripts.

Sincerely,

Dr Dena Howland
Associate Editor, Journal of Neuroscience Research

Dr Cristina Ghiani
Editor-in-Chief, Journal of Neuroscience Research


Associate Editor: Howland, Dena
Comments to the Author:
We are pleased to accept your manuscript for publication in the Journal of Neuroscience Research.


NIH GRANTEES: All NIH authors should also be aware that Wiley-Blackwell will submit manuscripts to the NIH Manuscript Submission system automatically. After their accepted manuscript files have been posted by Wiley-Blackwell to the NIH Manuscript Submission system, the corresponding author will receive an e-mail request from PubMed Central asking them to approve the upload for display on the PubMed Central system. Authors will need to provide approval to complete the process of deposition to PubMed Central. This is a requirement of their grant/affiliation.

P.S. You can help your research get the attention it deserves! Check out Wiley’s Promotion Guide for best-practice recommendations for promoting your work at www.wileyauthors.com/maximize.

_____________________________________________________________________________________
